# Energy-Efficient Collision-Free Machine/AGV Scheduling Using Vehicle Edge Intelligence

**DOI:** 10.3390/s24248044

**Published:** 2024-12-17

**Authors:** Zhengying Cai, Jingshu Du, Tianhao Huang, Zhuimeng Lu, Zeya Liu, Guoqiang Gong

**Affiliations:** Hubei Province Engineering Technology Research Center for Construction Quality Testing Equipments, College of Computer and Information Technology, China Three Gorges University, Yichang 443002, China

**Keywords:** automatic guided vehicles, energy efficient, collision-free scheduling, vehicle edge intelligence, artificial plant community algorithm

## Abstract

With the widespread use of autonomous guided vehicles (AGVs), avoiding collisions has become a challenging problem. Addressing the issue is not straightforward since production efficiency, collision avoidance, and energy consumption are conflicting factors. This paper proposes a novel edge computing method based on vehicle edge intelligence to solve the energy-efficient collision-free machine/AGV scheduling problem. First, a vehicle edge intelligence architecture was built, and the corresponding state transition diagrams for collision-free scheduling were developed. Second, the energy-efficient collision-free machine/AGV scheduling problem was modeled as a multi-objective function with electric capacity constraints, where production efficiency, collision prevention, and energy conservation were comprehensively considered. Third, an artificial plant community algorithm was explored based on the edge intelligence of AGVs. The proposed method utilizes a heuristic search and the swarm intelligence of multiple AGVs to realize energy-efficient collision-free scheduling and is suitable for deploying on embedded platforms for edge computing. Finally, a benchmark dataset was developed, and some benchmark experiments were conducted, where the results revealed that the proposed heuristic method could effectively instruct multiple automatic guided vehicles to avoid collisions with high energy efficiency.

## 1. Introduction

Due to the advantages of automatic guided vehicles (AGVs), they have obtained wide application in production systems [1], including various forms of production [2], smart logistics [3], manufacturing [4], transportation [5], etc. Now, more and more businesses implement all kinds of AGVs. The co-operation of multiple AGVs can further improve production efficiency and operating speed, averting human-made errors and fatigue risk [6]. AGVs are powered by batteries and use automatic guidance devices, such as sensors, magnetic strips, tracks, or lasers, to travel along planned paths. They are often equipped with safety protection and various auxiliary mechanisms, such as transfer and assembly mechanisms. Common AGVs include automotive chassis assembly AGVs, unmanned ground vehicles (UGVs) [7], heavy-duty AGVs, diesel engine assembly AGVs, gearbox-assembly AGVs, handling AGVs, intelligent inspection AGVs, logistics AGVs, and some unmanned surface vehicles (USVs) [8]. Promoting the machine/AGV system has gained widespread recognition in the industry to achieve greater production efficiency and enhance the business resilience of all sorts of industrial systems [9].

Due to the completely unmanned control of an AGV, the greatest challenge in machine/AGV systems is collision [10], especially when sensors fail, making them more prone to collision and safety accidents. Collision-free scheduling technologies can enhance the reliability and security of a machine/AGV system, which often integrates multiple sensor technologies, the Internet of Things (IoT), cloud centers, edge computing, and wireless networks [11]. Advanced technologies such as vehicle edge intelligence (VEI) have also been developed [12]. The VEI methods based on artificial intelligence (AI) have great potential for application in intelligent transportation systems, automatic robots, production, manufacturing, and so on [12]. AI technologies can integrate various sensors, IoT, cloud computing, and edge computing devices to build an intelligent cyber system that can efficiently detect the external environment, anticipate potential hazards, and further enhance system security and scalability [13,14].

Solving the collision-free scheduling problem (CSP) of a machine/AGV system requires energy consumption, such as sensor positioning, algorithm computation, and scheduling control. However, a machine/AGV system is often powered by limited-capacity batteries, so improving the energy efficiency of AGVs is also an important issue [15,16]. Therefore, the energy-efficient collision-free machine/AGV scheduling problem (ECMSP) for machine/AGV systems can be regarded as a typical multi-objective optimization problem for production efficiency, collision prevention, and energy conservation with battery capacity constraints [11]. Balancing the transportation efficiency, collision avoidance, and energy efficiency of machine/AGV systems simultaneously is a challenging task [17], as these objectives conflict, and it is difficult for traditional methods to solve such non-linear multi-objective problems. 

This paper focuses on the edge intelligent scheduling strategies in production systems, which are based on the artificial plant community (APC) algorithm [18,19]. A novel VEI architecture constitutes the core of a machine/AGV system for implementing collision prevention and energy conservation. Our contributions mainly include the following four aspects:

First, a VEI system was developed to improve and realize energy-efficient collision-free machine/AGV scheduling, where each AGV is an intelligent edge device, i.e., an edge AGV (EAGV). The VEI system includes a virtual planning layer and a physical optimization layer, and the corresponding state transition diagrams for collision-free scheduling were developed. Compared with the existing AGV optimization framework, VEI provides a new idea to solve similar challenges in the field of production management, that is, without additional software and hardware upgrades; it only integrates the limited edge computing capabilities of multiple vehicles to complete complex task solving.

Second, the energy-efficient collision-free machine/AGV scheduling problem was modeled as a multi-objective scheduling function with electric capacity constraints, where production efficiency, collision prevention, and energy conservation were comprehensively considered. A time window and a load balance index were added to the virtual planning layer, and the makespan and energy conservation indexes were added to the physical optimization layer.

Third, an APC algorithm was explored based on the edge computing capacity of AGVs, which utilizes the heuristic search and swarm learning mechanism of the machine/AGV system to realize energy-efficient collision-free scheduling. The control and computing architecture of the APC is decentralized and can be distributed in edge AGVs for fast computing.

Fourth, a benchmark dataset was developed based on a modified production workshop [11], and a series of benchmark experiments were conducted. The experiments verified the validity of the distributed VEI model to achieve energy-efficient collision avoidance, and our modified benchmark test can be employed for other similar tests.

The remainder of this paper is organized as follows. Section 2 reviews the state-of-the-art research. Section 3 illustrates a VEI architecture for a machine/AGV system. Section 4 covers the design of a multi-objective function for the energy-efficient collision-free machine/AGV scheduling problem. Section 5 shows the heuristic APC search algorithm to address the problem. Section 6 covers a series of benchmark tests for proactive collision avoidance and energy optimization, and Section 7 concludes this paper.

## 2. Background

In this section, two main kinds of related research are discussed: collision avoidance in machine/AGV systems and energy efficiency in multiple AGVs.

### 2.1. Collision Avoidance in Machine/AGV Systems

The concept of collision avoidance appeared closely after AGVs and is the main challenge of machine/AGV systems, where there are two main methods, i.e., the physical method and the virtual one. 

The physical method is used to improve the hardware and physical entities of AGVs, including various sensors, wireless communications, cloud servers, production systems, and control systems [20]. Modern AGVs widely integrate advanced hardware technologies and physical protocols, which in turn have also facilitated the promotion and application of AGVs [21]. With the aid of physical hardware, a machine/AGV system can greatly improve environmental perception, risk adaptation, self-organizing ability, and security. With the improvement in hardware performance, an AGV also has stronger computing and storage capabilities, providing a physical basis for edge computing and various software methods. The application of VEI in industrial systems provides a possible solution for collision avoidance of machine/AGV systems [14]. However, the cost of hardware methods is also relatively high, which can lead to more complex hardware structures. Once a sensor fails, hardware methods may lead to the failure of AGV obstacle avoidance [22].

The virtual method is used to improve service management and software scheduling to achieve efficient collision avoidance in a machine/AGV system, including speed control strategies [22,23], structural online control policies [24], pickup and delivery hybrid operations [25], integrated scheduling [26], and heuristic algorithms. This method depends on intelligent scheduling strategies and self-managed software on multiple physical AGV systems, ensuring the reliability of collision avoidance. The virtual method provided an intelligent architecture with self-organizing and adaptive capabilities for machine/AGV systems and can effectively realize timely collision avoidance by using all kinds of AI algorithms, including genetic algorithm (GA) [9,17], fuzzy logic (FL) [6], whale optimization algorithm (WOA) [6], ant colony optimization (ACO) [27], artificial bee colony (ABC) [28], particle swarm optimization (PSO) [29,30], A* algorithm [31], D* algorithm [32], deep Q-network (DQN) [25,31], and gray wolf optimization (GWO) [4]. When there is a hardware failure, the software approach may still be able to calculate the collisions that may occur in multiple AGVs and adopt the optimal collision avoidance strategies. 

Today, there are no longer purely physical or virtual methods, and more comprehensive methods that integrate the two types are being used. These methods can be applied in situations where high reliability is required to provide reliable and resilient operation in machine/AGV systems [17]. In this architecture, the entities in the physical layer include various intelligent AGVs. The real-time status of an intelligent AGV could be mapped as a virtual vehicle in the virtual layer, which can run optimized strategies to be implemented in the physical layer [33]. With the improvement in edge computing capability, AGVs are likely to run more complex anti-collision computing tasks. This composite solution may bring better collision avoidance strategies to AGVs.

### 2.2. Energy Efficiency in Multiple AGVs

In the machine/AGV system, the realization of the operation and scheduling depends on the capacity of the battery it carries and the speed of power consumption. Therefore, the energy efficiency of AGVs is another significant challenge to their operational sustainability and battery life. Energy efficiency plays an important role in the sustainable operation of multiple AGVs and improving battery life. The current research mainly includes route optimization methods and power consumption optimization methods.

The first optimization method for route optimization is to optimize scheduling jobs and routes to decrease total distance since the power consumption of the battery is closely related to total mileage [34]. Many researchers verified that a good route [35] and an optimization scheduling strategy [36] could decrease total mileage and reach the target with less energy consumption. Many heuristic route planning algorithms are employed to solve the energy efficiency problem in AGVs, such as ACO [27,34], invasive weeds optimization algorithm [37], and deep reinforcement learning (DRL) [13,38]. They often used a multi-agent system (MAS) [5,39] and hierarchical strategies to search for the optimal routes for AGVs with high energy efficiency, where the routes will be repeatedly re-planned according to the dynamic environment and battery power remaining [40]. However, complex algorithms require more computing and storage resources on multiple AGVs, which may not be feasible for real-time scheduling. 

The second optimization method for power consumption characteristics is based on the battery discharge curve because different driving behaviors can lead to changes in the power consumption curve, and aggressive driving behavior often results in a rapid decrease in battery life. For an AGV, the faster the speed, the higher the power consumption. Rapid acceleration or deceleration will also consume more power. Therefore, it is important to build a dynamic programming method based on charging and maintenance, which tries to maintain the AGV in a medium and constant-speed driving state as much as possible to reduce energy consumption during operation. In production systems, other factors may also lead to a reduction in AGV driving range, such as load, road smoothness, environmental conditions, air temperature, and traffic conditions. Heavy load and uneven road surfaces often consume more battery power. The reduction in ambient temperature can easily lead to a decrease in battery activity and remaining capacity. Traffic congestion and collisions can also lead to additional power consumption. 

As these two challenges are combined in AGVs, the energy-efficient collision-free machine/AGV scheduling problem is beginning to receive attention. On the one hand, collisions may lead to more energy consumption; on the other hand, poor energy management may lead to collisions. In terms of the energy efficiency of multi-AGVs, the use of dynamic optimization models is often necessary to complete the requested transportation missions collision-free [8]. However, complicated computation processes in the energy-efficient collision-free machine/AGV scheduling algorithm require high computing and storage capacity and reduce applicability in large-scale AGV systems.

### 2.3. Research Gap

In view of the above challenges in related areas, there are four issues.

First, the research on AGV collision avoidance and energy efficiency is often conducted independently [8]. Most AGVs use various sensors to identify obstacles and thereby re-plan routes to avoid collisions. Unbalanced cargo loads may affect resource allocation and route planning and will cause more congestion, collisions, and energy consumption. Traditional passive collision avoidance methods rarely consider energy efficiency, and the sensing ranges of radars or infrared sensors are limited. Once there is a signal failure or another fast-moving AGV, it is possible for this to lead to a collision or the need to consume more energy to re-plan routes to avoid collisions; that is, the collision avoidance problem and energy efficiency problem are closely interrelated; therefore, the spatial route distribution of machine/AGV systems may affect the effective solution of the problem.

Second, the use of traditional ITS architectures makes it difficult to solve the energy-efficient collision-free machine/AGV scheduling problem [41]. This type of problem often requires a complex computational process and requires high computational and storage capabilities, but there is high uncertainty in time-consuming computing tasks. Artificial intelligence technologies represented by deep learning have demonstrated strong advantages in solving such complex problems, but their requirements for computing platforms are also very high [25,31]. The moving range of multiple AGVs is very large, but the computing and storage capabilities of the embedded computing platforms used by AGVs are extremely limited. A large-scale machine/AGV system needs to respond quickly to a large number of anti-collision requests, which places high demands on the physical architecture and hardware facilities, increasing the difficulty of problem-solving. New attempts that employ the decentralized vehicle edge intelligence architecture of physical AGVs may be promising, but integrating edge computing and AI-driven AGV optimization methods is very rare.

Third, it is difficult to design efficient algorithms on embedded computing devices to solve the energy-efficient collision-free machine/AGV scheduling problem [42]. It is difficult to obtain optimal solutions when solving such large-scale AGV problems when using traditional algorithms, such as the Dijkstra algorithm [34] and the greedy algorithm. To decrease the computational cost and enhance the time performance of algorithms, spatial location methods and time-sensitive algorithms are often used to search for the shortest distance. The integration of multi-objective optimization with edge computing will be a promising direction to reduce computing time in large-scale AGV systems, but current research in this area is almost non-existent.

Fourth, the benchmark test dataset in this field is also very limited [8,11]. Test data on AGV collision avoidance and energy efficiency are required. The integration of the practical planning solution of machine/AGV systems, the unbalanced distribution of cargo loads, and energy-efficient collision-free scheduling should be taken into account simultaneously.

Therefore, this paper proposes a vehicle edge intelligence architecture for multiple AGVs to ensure energy-efficient collision avoidance. The VEI-based machine/AGV system has strong edge computing and storage capacity and can implement distributed algorithms at low cost.

## 3. A VEI-Based Architecture for the Machine/AGV System

In this section, we cover the introduction of VEI architecture into the energy-efficient collision-free machine/AGV system. Then, the state transition diagram that reflects the scheduling process and cargo balance is illustrated.

### 3.1. Characteristics of the VEI Architecture

**Figure 1:**
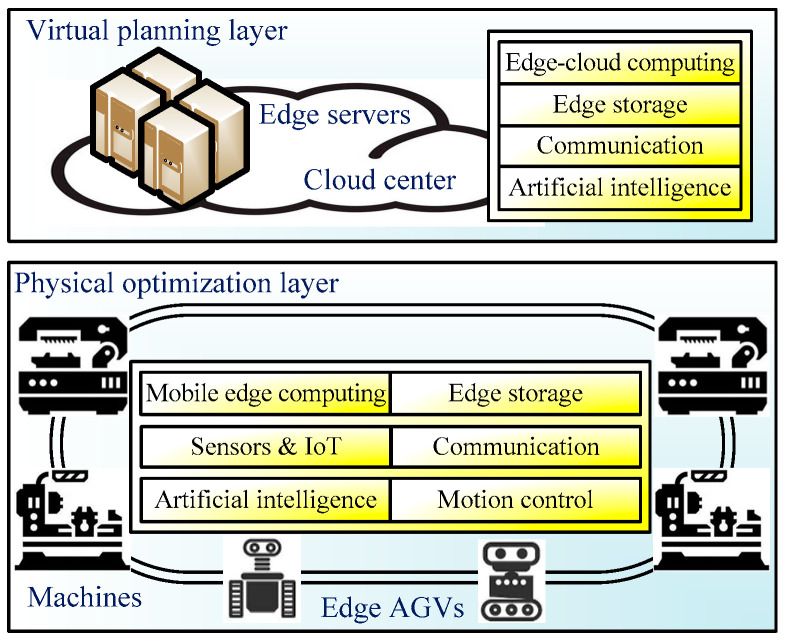
The VEI-based architecture for a machine/AGV system.

Figure 1 shows a VEI-based architecture for the machine/AGV system, which can be deployed in various production systems. It mainly consists of two layers: the virtual planning layer and the physical optimization layer.

The virtual planning layer (VPL) has a cloud center and numerous edge servers to provide high edge-cloud computing capacity, including data processing and route planning in the machine/AGV system. It plans the transportation routes of all AGVs according to the task states when they accept the user tasks. The primary function of the virtual planning layer is to pre-arrange the driving route to avoid collisions, balance the load on different routes, and optimize energy consumption before working on the edge AGVs. The edge servers can perform optimal scheduling algorithms according to the production tasks and the current battery state. Thus, the AGV entities in the physical optimization layer are mapped to virtual forms in the virtual planning layer to ensure efficient and secure scheduling. At the same time, the cloud center has the aid of human–computer interaction to accept human operating instructions and adjust task state information.

The physical optimization layer (POL) has a great number of machines and edge AGVs, which have mobile edge computing and edge storage capacity and are equipped with many sensors, IoT modules, communication and motion control modules, as well as artificial intelligence modules. The onboard processor of the AGV is an embedded processing center for edge computing and data processing and can offload some edge computing tasks to other edge AGVs or edge servers in the cloud center. Multiple AGVs can work together and undertake most of the edge computing tasks under the VEI architecture, including collision avoidance and energy saving. Multiple AGVs perceive the production environment through various sensors and IoT in real time, and they upload the localization information, task states, and vehicle states to the cloud center. 

The definitions and comparisons of VEI are shown in Table 1, including level, definition, role, attribute, function, architecture, computational ability, and storage capacity. Here, vehicle edge intelligence constitutes the swarm intelligence of edge servers and edge AGVs. The virtual planning layer serves as the software layer in the VEI architecture, while multiple edge AGVs in the physical optimization layer form the hardware layer. A physical machine/AGV in the VPL can be mapped as a virtual machine/AGV in the POL, and a virtual AGV in the POL can schedule a physical AGV in the VPL. Edge servers and edge AGVs have equal status in the VEI architecture and can offload computing tasks from each other. However, edge servers can utilize their more powerful computing power to perform more difficult computing tasks that edge AGVs cannot perform. Various communication devices connect the virtual layer and the physical layer and provide reliable and secure links between edge devices. By offloading parts of computing tasks onto other edge devices, the VEI architecture is suitable for solving the energy-efficient collision avoidance problem of machine/AGV systems, and the computing cost and delay can be greatly reduced.

The proposed vehicle edge intelligence can improve traditional production systems with better computing power and business flexibility for solving energy-efficient collision-free machine/AGV scheduling problems in multiple AGVs, and the computing power will increase as the number of AGVs increases. The virtual planning layer selects a suitable AGV route network according to cargo volume and load balance, and the physical optimization layer realizes collision avoidance and energy conservation in a production system. The engineering applications of the VEI architecture for a machine/AGV system are based on the state transition diagrams in the following sections.

### 3.2. The State Transition Diagram for the VPL

For the VEI-based architecture, the accurate state transition of the production tasks and machines/AGVs is necessary. The states of the production tasks and machines/AGVs should be detected by a VEI-based machine/AGV system and are universal and applicable to all kinds of production systems. Based on the state transition, the virtual planning layer can perceive machine/AGV information and optimize the scheduling schemes to diminish collision and energy consumption. Then, the physical optimization layer can further optimize transportation routes and be collision-free in real time.

**Figure 2:**
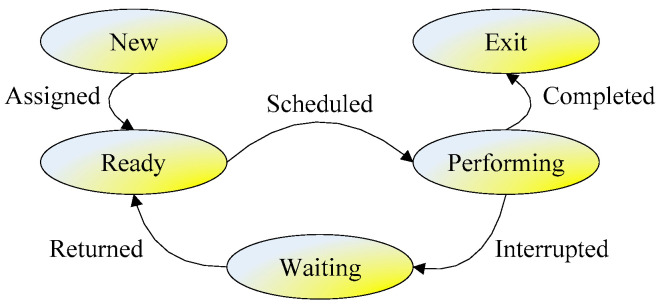
The task state transition diagram.

To illustrate the production task scheduling process in VPL, a task state transition diagram is defined for AGV edge computing in Figure 2. There are five production task states corresponding to five ellipses in the figure, and the five arrows between the states determine the state transition process of tasks; the text on the arrows means the action to trigger the task state transition. The five states are illustrated as follows:

- New: This is the first state. If a task requests a machine/AGV, it will be created at first and will wait for an assignment;
- Ready: If a new task is assigned to an idle machine/AGV, it will be ready before performing a task. Then, the machine/AGV is occupied by the task and cannot be used for other purposes;
- Performing: If a task that is ready is scheduled, then the task can be performed on a machine/AGV;
- Waiting: When a task is being performed on an AGV, an interruption, i.e., a collision, will break off performance of the task before its completion, and the interrupted task will be set as a waiting state. If the interruption is completed, the waiting task will be returned to the ready state;
- Exit: Whenever a task is completed, it will exit the VEI system, and then the AGV is idle again, waiting for the next task schedule.

### 3.3. The State Transition Diagram for the POL

**Figure 3:**
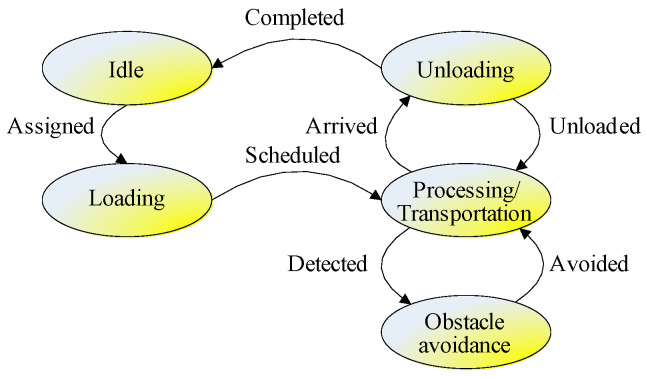
The machine/AGV state transition diagram.

To illustrate the machine/AGV scheduling process in POL, a machine/AGV state transition diagram is explored for AGV edge computing, as shown in Figure 3. There are five machine/AGV states in total, corresponding to five ellipses in the figure. The arrows between the states indicate the state transition process of machines/AGVs, and the text on the arrows indicates the action to trigger the machine/AGV state transition. The five states are illustrated as follows:

- Idle: This is an initial state where a machine/AGV is not assigned to any tasks. Only an idle machine/AGV can be assigned to a task;
- Loading: When a machine/AGV is assigned to a task, the idle machine/AGV is occupied and begins to load the cargo;
- Processing/Transportation: If a machine/AGV has loaded cargo for an assigned task, then it begins to process/transport the cargo;
- Obstacle avoidance: Whenever an obstacle is detected, the machine/AGV begins to adopt different strategies to avoid obstacles, including deceleration, acceleration, stop, and detour. When the obstacle is avoided, the machine/AGV will continue processing/transportation;
- Unloading: After the machine/AGV finishes processing/transportation, the machine/AGV will unload the cargo. If all cargo is unloaded and all tasks are completed, the machine/AGV will be in an idle state again and can be assigned to the next production task.

## 4. Problem Modeling

The key to the energy-efficient collision-free problem is to balance production efficiency, collision avoidance, and energy consumption. Therefore, the proposed solving method turns out to be active, as the virtual planning layer can detect the occurrence of congestion and collision early. This is helpful in reducing congestion and collision in a production system by considering the cargo balance and energy consumption on different routes. 

To simplify problem modeling, we propose the following assumptions:

1. All production tasks are assumed to be independent without considering their inter-relationships. There is no preference for the same production task on different machines or AGVs;
2. All machines are assumed to be independent without considering their correlation with each other or their preferences for cargo;
3. All AGVs are assumed to be independent without considering their correlation with each other or the differences in the loading of different cargo;
4. The impact of the volume and shape of the cargo on processing and transportation time are not considered;
5. The impact of working hours on the power changes in machines and AGVs are not considered. Sufficient power supply for machines and AGVs is assumed.

### 4.1. The Notations and Explanations

The notations and explanations used in the following sections are shown in Table 2.

In a production system, there is a set of machines defined as follows:

(1) $$N=\{N_{1},N_{2},\ldots ,N_{i},N_{j},\ldots ,N_{max}\}$$

A set of edges between these machines can be expressed as

(2) $$E=\{e_{ij}|i,j=1,2,\ldots ,N_{max}\}$$

A set of cargo gives the load of each cargo.

(3) $$C=\{w_{1},w_{2},\ldots ,w_{c},\ldots ,w_{C_{max}}\}$$

A set of AGVs is

(4) $$V=\{1,2,\ldots ,v,\ldots ,V_{max}\}$$

A production task T can be given as

(5) $$T=\left\{T_{1},T_{2},\ldots ,T_{max}\right\}$$

Hence, an energy-efficient collision-free machine/AGV scheduling problem can be described as a machine set, N, an edge set, E, a cargo set, C, and an AGV set, V, that is, ECMSP={N,E,C,V}. The solution to ECMSP can be described as an assignment to arrange a set of machines/AGVs to transport a series of cargo through different nodes and edges with low energy consumption and collision avoidance.

There are two nodes with the co-ordinates $i\left(x_{i},y_{i}\right)$ and $j\left(x_{j},y_{j}\right)$, and the distance between them can be calculated as

(6) $$d_{ij}=\sqrt{{\left(x_{i}-x_{j}\right)}^{2}+{\left(y_{i}-y_{j}\right)}^{2}}$$

### 4.2. Task Planning in the VPL

The task planning in the VPL is the preliminary planning of the transportation routes for a variety of AGVs, including optimizing and balancing the cargo load on different routes, reducing unnecessary AGV transportation routes, ensuring that each production task can be completed smoothly, and detecting possible collisions in advance. 

Based on the four rules above, the objective function of task planning in the VPL can be built.

The first objective of task planning in the VPL is to obtain the total load $w_{\Sigma }$ of all cargo in a task. The production task includes a set of cargo, C, and the load of cargo c is $w_{c}$. However, not all cargo can be loaded by an AGV in a task, and the load of AGV v is $w_{v}$. Then, the total load of all cargo in a task can be calculated as

(7) $$w_{\Sigma }=\sum_{v=1}^{V_{max}}w_{v}$$

After that, the cargo is assigned to several AGVs, and the selection bit of cargo c on AGV v is $\lambda_{vc}$. Hence, the load of AGV v can be calculated as

(8) $$w_{v}=\sum_{c=1}^{C_{max}}{\lambda_{vc}w}_{c}$$

Equation (8) indicates that if an AGV cannot complete the cargo transportation at once, it needs to run more times or increase the number of AGVs, which will increase the risk of congestion and collision.

The second objective of task planning in the VPL is the load balance of all AGVs in a task. According to the maximum load capacity $w_{v}^{max}$ of AGV v, the load factor $L_{v}$ can be calculated as

(9) $$L_{v}=w_{v}/w_{v}^{max}$$

Equation (9) indicates that if the cargo of multiple AGVs is unbalanced, it will lead to an increase in transportation routes, as well as an increase in the possibility of congestion and collisions. The larger this ratio, $L_{v}$, the heavier the cargo loaded by the AGV, and the higher the energy consumption may be; in contrast, energy consumption is low. Normally, this value should not exceed 1.0; otherwise, this represents overloading and will result in greater energy consumption.

Then, the maximum load factor $L_{v}^{max}$ and the minimum load factor $L_{v}^{min}$ of all AGVs can be calculated.

(10) $$\left\{\begin{array}{c} L_{v}^{max}=\max\{L_{v}|{1\leq v\leq V}_{max}\}\\ L_{v}^{min}=\min\{L_{v}|{1\leq v\leq V}_{max}\} \end{array}\right.$$

$L_{v}^{max}$ in the first line of Equation (10) represents the AGV with the heaviest load, and $L_{v}^{min}$ in the second line of Equation (10) denotes the AGV with the lightest load, so their deviation $\left(L_{v}^{max}-L_{v}^{min}\right)$ can be used to describe the degree of load balance of all AGVs. Equation (10) indicates that if the cargo of an AGV is too heavy, it can lead to rapid power consumption. Otherwise, AGVs with a cargo that is too light can also lead to unnecessary energy waste. 

The third objective of task planning in the VPL is the total number of AGVs assigned to a task. The real number of AGVs is decided by the assignment, that is,

(11) $$V_{max}^{\prime }=\sum_{c=1}^{C_{max}}\lambda_{vc}$$

Therefore, according to Equations (7), (10) and (11), the objective function of the task planning in the VPL can be described as follows:

(12) $${Obj}_{VPL}=\{\max\left(w_{\Sigma }\right),\min\left(L_{v}^{max}-L_{v}^{min}\right),\min(V_{max}^{\prime })\}$$

(13) $$st:\;{c\leq C}_{max},\;{v\leq V}_{max},w_{v}\leq w_{v}^{max},{0<L}_{v}\leq 1$$

The objective function in Equation (13) includes three conflicting objectives: the total load $w_{\Sigma }$ of all cargo, the cargo balance $\left(L_{v}^{max}-L_{v}^{min}\right)$, and the real number $V_{max}^{\prime }$ of AGVs. That is, the objective of the task planning in the VPL is to transport more cargo using fewer AGVs and fewer routes, where the cargo loads of different AGVs do not differ significantly. This is not an easy task, as transporting more cargo often requires more AGVs, and using fewer AGVs will also constrain the maximum transportation volume.

The constraints in Equation (13) are composed of four parts. The first part means the limitation $C_{max}$ of the number of cargoes in a production task. The second part constrains the total number of vehicles $V_{max}$. The third part limits the maximum load capacity $w_{v}^{max}$ of AGV v. The last constraint defines the load factor $L_{v}$ (not to overload).

### 4.3. AGV Optimization in the POL

The AGV optimization in the POL further optimizes the driving behavior of different AGVs, including maintaining production efficiency, an energy-efficient uniform speed driving state, reducing unnecessary deceleration and acceleration, decreasing the number and angle of turns, selecting the optimal route to complete production tasks, and adopting the optimal energy-saving obstacle avoidance route and speed.

Based on the four rules above, the objective function of the AGV optimization in the POL can be constructed. In a production system, there is a task set, $T=\left\{T_{1},T_{2},\ldots ,T_{max}\right\}$, a node set,$\;N=\{N_{1},N_{2},\ldots ,N_{max}$}, an edge set, $E=\{e_{ij}|i,j=1,2,\ldots ,N_{max}\}$, the cargo set, $C=\{1,2,\ldots ,w_{C_{max}}$}, and an AGV set, $V=\{1,2,\ldots ,V_{max}$}. The complete route from the start node i=a to the end node j=b is described as an arc set $\Lambda =\left\{\lambda_{vij}|i,j\in N\right\}$, where i,j are the nodes at both ends of the arc, and i is the start node. $\lambda_{vij}$ sequentially comprises all arcs on the route. For arc $\lambda_{vij}\in E$, the value can be defined as $\lambda_{vij}=\left\{0,1\right\}$, where 1 and 0 mean whether the arc is selected in the planned route, respectively.

The first main objective of the AGV optimization in the POL is makespan, $t_{\Sigma }$, which means production efficiency regarding transporting all cargo to a destination and processing them in a short time. The unit time window $t_{vij}\in \left[t_{vi}^{enter},t_{vj}^{leave}\right]$ of AGV *v* on edge $e_{ij}$ can be calculated as follows, where $t_{vi}^{enter}$ and $t_{vj}^{leave}$ are the time points of entering and leaving edge $e_{ij}$, respectively:

(14) $$t_{vij}=d_{ij}/s_{v}\left(t\right)$$

That is, the time window is determined by the driving speed $s_{v}$ of AGV *v* and the distance between two nodes $i\left(x_{i},y_{i}\right)$ and $j\left(x_{j},y_{j}\right)$. Equation (14) denotes that the uniform linear motion of AGVs is the most energy efficient. Hence, the longest travel time, $t_{v}$, of an AGV can be expressed as

(15) $$t_{v}=\max_{v}(\sum_{i=a}^{j=b}t_{vij}\lambda_{vij})$$

Based on Equation (15), the total time cost of all AGVs can be determined by using the maximum value of the total time costs of all AGVs, that is, the longest travel time of AGVs. 

The makespan of a production task still needs to consider the processing time $t_{ci}^{proce}$ on machine i, which is decided by the sum of the processing time of all cargo on node i.

(16) $$t_{i}=\sum_{c=1}^{C_{max}}t_{ci}^{proce}{\lambda_{vc}\lambda }_{vij}$$

The makespan task depends on the maximum value of the sum of the transportation time, $t_{v}$, on all AGVs and processing time, $t_{i}$, on all machines, that is,

(17) $$t_{\Sigma }=\max_{i}\{\sum_{v}t_{v}{+t}_{i}\}$$

The second main objective of the AGV optimization in the POL is to reduce the total energy consumption, $e_{\Sigma }$, including AGV energy consumption and machine energy consumption. 

The first part is AGV energy consumption, $e_{v}$, which can be calculated by using driving power and driving time. For a route section, $e_{i,j}$, from node i to j, the time of entering node i is $t_{vi}^{enter}$, the time of leaving node j is $t_{vj}^{leave}$, the driving speed at time t is $s_{v}(t)$, and the driving power at time t is $p_{v}^{driv}\left(t\right)$. In addition, energy consumption includes the stop, turning, and acceleration energy consumptions, corresponding to time $t_{vij}^{stop}$, $t_{vij}^{turn}$, and $t_{vij}^{acce}$, respectively. Therefore, the energy consumption, $e_{vij}$, of the AGV v on that route section can be calculated as

(18) $$e_{vij}=p_{v}^{driv}\left(t\right)\left(t_{vj}^{leave}-t_{vi}^{enter}-t_{vij}^{stop}-t_{vij}^{turn}-t_{vij}^{acce}\right)+p_{v}^{stop}\left(t\right)t_{vij}^{stop}+p_{v}^{turn}\left(t\right)t_{vij}^{turn}+p_{v}^{acce}\left(t\right)t_{vij}^{acce}$$

The energy consumption in Equation (18) includes four parts, i.e., the driving energy consumption, stop energy consumption, turning energy consumption, and acceleration/deceleration energy consumption. In the process of collision avoidance, the AGVs have to perform various maneuvers, including straight ahead, deceleration, detour, stop, and acceleration/deceleration. Equation (18) indicates that if the AGV obstacle avoidance route is too long or if two or more AGVs avoid obstacles simultaneously, it will increase unnecessary energy loss.

The driving power, $p_{v}^{driv}$, stop power, $p_{v}^{stop}$, turning power, $p_{v}^{turn}$, and acceleration power, $p_{v}^{acce}$, of AGV *v* depend on the driving force, $F_{v}^{driv}$, stop force, $F_{v}^{stop}$, turning force, $F_{v}^{turn}$, and acceleration force, $F_{v}^{acce}$, of AGV *v*, respectively. We have

(19) $$\left\{\begin{array}{c} p_{v}^{driv}=F_{v}^{driv}(L_{v})s_{v}\left(t\right)\\ p_{v}^{stop}=F_{v}^{stop}(L_{v})s_{v}\left(t\right)\\ p_{v}^{turn}=F_{v}^{turn}(L_{v})s_{v}\left(t\right)\\ p_{v}^{acce}=F_{v}^{acce}(L_{v})s_{v}\left(t\right) \end{array}\right.$$

The driving power, $p_{v}^{driv}$, stop power, $p_{v}^{stop}$, turning power, $p_{v}^{turn}$, and acceleration power, $p_{v}^{acce}$, of AGV *v* are simultaneously affected by the speed, $s_{v}\left(t\right)$, and force of the AGV. The faster the speed is, the greater the required force is, resulting in greater energy consumption. At the same time, the driving force, $F_{v}^{driv}$, stop force, $F_{v}^{stop}$, turning force, $F_{v}^{turn}$, and acceleration force, $F_{v}^{acce}$, of AGV *v* are affected by the load factor, $L_{v}$, of the AGV. The greater the cargo load is, the greater the force is. Equation (19) describes the fact that the frequent acceleration and deceleration of AGVs will increase energy consumption, and large angle turning and frequent turning (of AGVs) will increase energy consumption.

According to Equation (19), the energy consumption, $e_{v}$, of AGV v can be obtained as follows:

(20) $$e_{v}=\sum_{i,j=1}^{N_{max}}e_{vij}$$

The second part is machine energy consumption, $e_{i}$, which can be calculated by using the processing power and the processing time. According to the processing time, $t_{ci}^{proce}$, when cargo c is on node i, and the processing power, $p_{ci}^{proce}$, when cargo c is on node i, we can obtain the processing energy consumption, $e_{ci}$, of node i. We have

(21) $$e_{ci}=p_{ci}^{proce}t_{ci}^{proce}$$

Then, machine energy consumption, $e_{i}$, can be given as follows:

(22) $$e_{i}=\sum_{c=1}^{C_{max}}e_{ci}$$

According to the energy consumption, $e_{v}$, of AGV v in Equation (20) and the machine energy consumption, $e_{i}$, in Equation (22), we have the total energy consumption, $e_{\Sigma }$, as follows:

(23) $$e_{\Sigma }=\sum_{v=1}^{V_{max}}e_{v}+\sum_{i=1}^{N_{max}}e_{i}$$

The third major objective of AGV optimization in the POL is the total collision time, $t_{\Sigma }^{colli}$.

(24) $$t_{\Sigma }^{colli}=\sum_{i=1}^{N_{max}}t_{vi}^{colli}$$

where the collision time $t_{vi}^{colli}$ on node i can be calculated by using the overlapping time of the different AGVs staying on the same node. We have

(25) $$t_{vi}^{colli}=\sum_{v=1}^{V_{max}-1}\left|t_{(v+1)i}^{enter}-t_{vi}^{leave}\right|$$

The fourth main objective of AGV optimization in the POL is to balance the routes of AGVs. According to the distance in Equation (6), the total distance, $d_{Rv}$, of route $R_{v}$ can be given as follows:

(26) $$d_{Rv}=\sum_{i=1}^{i=N_{max}}\sum_{j=1}^{j=N_{max}}\left(\lambda_{vij}d_{ij}\right)$$

According to Equation (26), the total distances of the longest route and the shortest route can be calculated. Their deviation can also be obtained as follows:

(27) $$d_{Rv}^{balan}=\max_{v}\left\{d_{Rv}\right\}-\min_{v}\{d_{Rv}\}$$

The first part in Equation (27) represents the longest AGV route, and the second part denotes the shortest AGV route, so their deviation can be used to describe the degree of route balance.

Therefore, according to Equations (17), (23), (24) and (27), the objective function of the AGV optimization in the POL can be described as follows:

(28) $${Obj}_{POL}=\{\min\left(t_{\Sigma }\right),\min\left(e_{\Sigma }\right),\min\left(t_{\Sigma }^{colli}\right),\max(d_{Rv}^{balan})\}$$

(29) $$st:\;e_{v}\leq B_{v}^{max}$$

(30) $$t_{vi}^{leave}\geq t_{vi}^{enter}+t_{vi}^{proce},$$

(31) $${\forall t_{vij}\lambda_{vij}\geq 0,t}_{vij}\in \left[t_{vi}^{enter},t_{vj}^{leave}\right],$$

(32) $$\sum_{j}\lambda_{vaj}-\sum_{j}\lambda_{vja}=1\left(j\in N,j\neq a\right),$$

(33) $$\sum_{i,j}\lambda_{vij}-\sum_{i,j}\lambda_{vji}=0\left(i\in N,i\neq a,i\neq b\right),$$

(34) $$\sum_{j}\lambda_{vbj}-\sum_{j}\lambda_{vjb}=-1\left(j\in N,j\neq b\right),$$

(35) $$s_{v}\leq s_{v}^{max}$$

(36) $$w_{v}\leq w_{v}^{max}$$

The objective function in Equation (28) contains four conflicting objectives, i.e., the production efficiency (makespan), $t_{\Sigma }$, total energy consumption, $e_{\Sigma }$, total collision time, $t_{\Sigma }^{colli}$, and the route balance index, $d_{Rv}^{balan}$, of all AGVs. That is, the objective of the AGV optimization in the POL is to transport cargo as fast as possible using less energy consumption, where the length of the driving routes for all AGVs is not significantly different. This is not an easy task since a shorter makespan requires faster transportation and more energy consumption, and there are various collision-free maneuvers to consume energy and increase route length.

The constraint in Equation (29) is such that it constrains the amount of energy, $e_{v}$, consumed by an AGV, which cannot exceed its battery capacity, $B_{v}^{max}$.

The constraint in Equation (30) states that the limitation of the leave time window $t_{vi}^{leave}$ should not be earlier than the sum of the enter time, $t_{vi}^{enter}$, and process time, $t_{vi}^{proce}$, on node i.

The constraint in Equation (31) defines the non-negativity of the time window $t_{vij}$ of each arc with the total time cost, $t_{v}$, and indicates the limitation of the unit time window $t_{vij}$ should be on the route from i to j.

The constraints in Equations (32)–(34) limit the value of $\lambda_{vij}$ to be in the range of 0 to 1. Equation (32) guarantees only one start node. Equation (33) ensures a continuous path. Equation (34) makes sure that there is only one end node or one exit.

Equation (35) constrains the maximum driving speed of AGV *v*. Equation (36) limits the maximum load capacity, $w_{v}^{max}$, of AGV v. At the same time, Equations (35) and (36) jointly limit the driving power, $p_{v}^{driv}$, stop power, $p_{v}^{stop}$, turning power, $p_{v}^{turn}$, and acceleration power, $p_{v}^{acce}$, according to Equation (19).

As we can see from the objective functions of the energy-efficient collision-free machine/AGV scheduling problem in Equations (12) and (28), there are several conflicting objectives. It is very difficult to solve the multi-objective functions for traditional algorithms, so a novel artificial plant community algorithm is introduced in the following section.

## 5. APC-Based Edge Computing

This section covers the development of an artificial plant community algorithm to implement heuristic edge computing. The APC algorithm proposed in [18,19] has few parameters and low hardware requirements. Hence, it can easily be deployed on edge AGVs and is suitable for scalable population sizes. Through the swarm intelligence of a large number of AGVs, a powerful edge computing capability can be obtained to help us search for the optimal solution to the energy-efficient collision-free machine/AGV scheduling problem.

### 5.1. Methodologies

**Figure 4:**
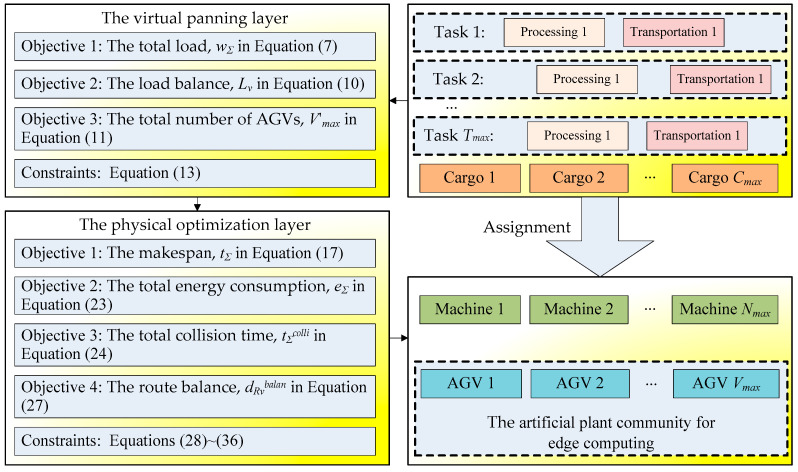
A schematic diagram of the APC-based edge computing framework.

A schematic diagram of the APC-based edge computing framework is shown in Figure 4, showing how the virtual planning and physical optimization layers interact to achieve conflict-free scheduling; this illustrates the relationships between the variables, objectives, and constraints.

For APC engineering applications in vehicle edge intelligence architecture, there are the following methodologies:

First, an artificial plant community is composed of a great number of individual plants and can be distributed on many edge AGVs. The APC algorithm has three basic operations, i.e., seeding, growing, and fruiting. All three operations can be realized by simple and/or/not operations on embedded platforms and are suitable for edge AGVs. The edge servers and the edge AGVs have the same status, and any failure will not affect the entire edge computing task. Therefore, they can offload computing tasks onto each other.

Second, one or more artificial plant individuals can be deployed on each edge device for edge computing. The edge servers in the cloud center usually undertake more complex and time-consuming computational tasks, i.e., select a suitable fitness function. Each edge AGV can load the fitness function defined in the edge servers and decide the life or death of the artificial plant individuals, where the APC individuals with low fitness die, whereas those with high fitness survive. If the fitness function is defined by the energy-efficient collision-free machine/AGV scheduling problem, the artificial plant individuals on edge AGVs will search for the optimal solution after tens of iterative calculations.

Third, there is no central control in the APC algorithm and edge computing. The artificial plant individual is randomly produced on each edge AGV or edge server, and it separately searches for feasible solutions to energy-efficient collision-free machine/AGV scheduling problems. Therefore, each edge AGV can perform better in the dynamic transportation and collision environment and can join or leave the VEI system at any time. The failure of one or more edge AGVs will not affect the solving process of the whole APC. The edge servers in the cloud center and the distributed edge AGVs have equal status and can mutually offload computing tasks. This decentralized VEI architecture has good business flexibility and scalable population size, and more edge AGVs will improve its computational ability.

Fourth, APC-based edge computing is a heuristic search method and solves problems by using iterative computation on edge AGVs or edge servers. During the solving process, the population size of the APC is variable and determined by a fitness function, i.e., Equation (12) or (28). Each edge AGV can share and interchange information with each other to select the optimal solution to the energy-efficient collision-free machine/AGV scheduling problem. The artificial plant individuals with high fitness can survive, but other individuals with low fitness will die, so the population size decreases. 

### 5.2. Solution Steps of the APC

Based on the previous section, this section illustrates the solution steps of the APC algorithm in detail. The whole solving process includes five main steps, i.e., initialization, seeding, growing, fruiting, and end justification. In different solving steps, each artificial plant individual has three forms, i.e., a seed, an individual, and a fruit. 

Step 1: Initialization

This step initializes three parts of the main parameters, including the parameters of the energy-efficient collision-free machine/AGV scheduling problem, the parameters of the APC, and the parameters of the solving system.

The parameters of the energy-efficient collision-free machine/AGV scheduling problem include a production task, $T=\left\{T_{1},T_{2},\ldots ,T_{max}\right\}$, the machine set, $N=\{N_{i}$}, the edge set, $E=\{e_{ij}\}$, the cargo set, $C=\{w_{c}$}, the AGV set, V={v}, the total number of nodes, $N_{max}$, the total number of AGVs, $V_{max}$, the total number of cargoes, $C_{max}$, the maximum load capacity, $w_{v}^{max}$ of AGV v, the maximum driving speed $s_{v}^{max}$ of AGV *v*, and the battery capacity, $B_{v}^{max}$, of AGV v.

The parameters of the APC involve the population size, S, the selection bits of a cargo set, {$\lambda_{vc}\}$, the selection bits {$\lambda_{vij}\}$ of an AGV set, the seeding probability, $p_{seed}$, the growing probability, $p_{grow}$, and the fruiting probability, $p_{fruit}$. Then, an artificial plant community can be defined as a solution set, $X=\{x_{1},x_{2},\ldots ,x_{i},\ldots ,x_{S}\}$, where $x_{i}$ = {$\lambda_{vc}\}$ in the VPL, or $x_{i}$ = {$\lambda_{vij}\}$ in the POL. The VPL objective function, ${Obj}_{VPL}$, in Equation (12), or the POL objective function, ${Obj}_{POL}$, in Equation (28), can be selected to construct a fitness function, fit, of APC.

(37) $$fit=Con-({Obj}_{VPL}\;or{\;Obj}_{POL})$$

where Con is a positive constant slightly larger than the maximum value of the objective function ${Obj}_{VPL}\;or{\;Obj}_{POL}$, ensuring a positive fitness value. Hence, the fitness function, fit, in Equation (37) can be used to select the best artificial plant individuals, where the higher the fitness, fit, the easier it is for artificial plant individuals to survive.

The parameters of the solving system need to set the maximum number of iterations, ${Ite}_{max}$, and the error threshold, $e_{th}$. In addition, the iteration counter, Ite, requires to be reset to zero.

Step 2. Seeding Of APC

The seeding process is a random search of the APC, where all artificial plant individuals are randomly distributed seeds in the whole solution space. This step provides a global search capability for an energy-efficient collision-free machine/AGV scheduling problem, which is determined by a seeding probability, $p_{seed}$, and is in the range of [0, 1], usually greater than 0.5. The seeds generated in the seeding step have two cases. 

The first seeding case takes place in the first iteration. After initialization, no individual or fruit has yet been produced, and the APC with a population size of S will be randomly produced in all edge AGVs, and the initial seeds are fully random. The solution of the APC algorithm is determined by the swarm learning process and is independent of the initial state of the seeds.

The second seeding case occurs in the following iterations. In this case, there are fruits with a population size of S produced in the previous fruiting steps to be selected as the seeds, and at the same time, several random seeds are generated with a population size of $p_{seed}\times S$. The best fruits and the random seeds constitute a new generation of seeds with a greater population size of $(1+p_{seed})\times S$. 

In the seeding step, the artificial plant seed, $x_{i}$, is encoded as a binary string for AGV assignment. In the virtual planning layer, the artificial plant individual, $x_{i}$, is encoded to assign all cargo to different AGVs and search for an optimal assignment for each AGV, as shown in Equation (38).

(38) $$x_{i}=\{\lambda_{vc}\}$$

In the physical optimization layer, the artificial plant individual, $x_{i}$, is encoded to assign all AGVs to different routes and to avoid collision, as shown in Equation (39).

(39) $$x_{i}=\{\lambda_{vij}\}$$

Whether in (38) or (39), each bit of $x_{i}$ denotes the binary selection of the corresponding feasible solution. A binary bit of $x_{i}$ = 1 indicates that cargo $w_{c}$, AGV v, or node i is selected as the candidate solution, but a binary bit of $x_{i}$ = 0 represents that cargo $w_{c}$, AGV v, or node i is removed from the candidate solution.

Step 3. Growing of the APC

The growing step can help the edge AGVs to choose the optimal solutions by using the growing probability, $p_{grow}$, and the growing population size of the APC decreases to a small value of $p_{grow}\times S$, while the death population size is (1 − $p_{grow})\times S$. $p_{grow}$ is in the range of [0, 1] and is usually greater than 0.5. In the growing stage, there is no artificial plant individual on some edge AGVs with a population size of (1 − $p_{grow})\times S$, but the best solutions survive on other edge AGVs with a population size of $p_{grow}\times S$. 

According to the fitness function, fit, in Equation (37), the solutions that survived, $x_{i}^{*}$, in the growing step are given as follows:

(40) $$x_{i}^{*}=\{x_{i}^{*}|fit\left({x_{i}^{*}\in p}_{grow}S\right)\geq fit({x_{i}\in (1-p}_{grow})S)\}$$

The growing probability, $p_{grow}$, plays an important role in the convergence capability of the APC. The smaller the growing probability, $p_{grow}$ is, the stronger the convergence capability is, but the weaker the global search capability is. This may even lead to a premature convergence to the local optima. In contrast, the greater the growing probability, $p_{grow}$, is, the slower the convergence is, and the stronger the global search capability is. 

Of these, the plant individual with the highest fitness is the most important elite in the whole solving process. According to the growing individual $x_{i}^{*}$ in Equation (40), the best one can be chosen as $x_{i}^{\#}$ in Equation (41).

(41) $$x_{i}^{\#}(t)=\{x_{i}^{\#}(t-1)|fit\left(x_{i}^{\#}\right)=\max[fit\left(x_{i}^{*}\right)]\}$$

To better apply the APC algorithm to the embedded platform of edge computing devices, a group sort is developed to help us focus on the best solution and implement quick selection. Each time, the group sort method randomly separates the seeds into $p_{grow}\times S$ groups, where each group has only two or three seeds. After that, each group chooses the best one from two or three seeds, so just one or two comparisons are required in each group. It can be calculated that the time performance and space performance of the group sort are O(S) and O(S), respectively, where its time and space performance are linearly related to the problem scale. Hence, the group sort can be easily deployed in distributed edge AGVs to efficiently search for the best $p_{grow}\times S$ of seeds in Equation (40) to grow and make sure that the best solution is obtained in Equation (41).

Step 4. Fruiting of the APC

The fruiting process is determined by a fruiting probability, $p_{fruit}$, which decides how great a proportion of parental information is well preserved in the next generation of fruits. An offspring fruit can learn most information from the main parent by using a fruiting probability, $p_{fruit}$, which is in the range of [0, 1] and is usually greater than 0.5; the offspring can learn the other portion of information from the other parents by using probability ${(1-p}_{fruit})$. The higher the fruiting probability, $p_{fruit}$, is, the stronger the ability for self-learning and the smaller the generation gap. In contrast, the smaller the fruiting probability, $p_{fruit}$, is, the stronger the ability for social learning, and the greater the generation gap. Therefore, a greater $p_{fruit}$ can maintain more information in excellent individuals and accelerate convergence performance. In contrast, a smaller $p_{fruit}$ makes it easier to destroy the information in excellent individuals and is less likely to converge to locally optimal solutions.

There are two ways to bear fruit in the fruiting step:

The first method is parthenogenesis, where a fruit is fully generated from the best individual, $x_{i}^{\#}$, with the highest fitness. This is a completely identical offspring with no social learning information. This method does not produce new solutions but just preserves the most important solution in the search process, as shown in Equation (42).

(42) $$x_{i}^{\prime }(t)=x_{i}^{\#}(t-1)$$

In social learning, the cross factor, $C_{fruit}$, determines how many parents collectively produce a child each time. If $C_{fruit}=2$, two individuals are involved in producing a fruit each time, and each fruit contains the information of two parents. This case has a certain social learning capability, and the calculation is also simple. Assuming there are two parents $x_{i}=\{x_{i1},\ldots ,x_{ij},x_{i\left(j+1\right)},\ldots \}$ and $y_{i}=\{y_{i1},\ldots ,y_{ij},y_{i\left(j+1\right)},\ldots \}$, they can produce a fruit, as shown in Equation (43).

(43) $$x_{i}^{\prime }(t)=\{x_{i1},x_{i2},\ldots ,x_{ij}{,y}_{i\left(j+1\right)},\ldots \}\;\mathrm{Or}\;y_{i}^{\prime }(t)=\{y_{i1},y_{i2},\ldots ,y_{ij}{,x}_{i\left(j+1\right)},\ldots \}$$

If $C_{fruit}\geq 3$, three or more individuals are involved in generating a fruit each time, and each fruit contains three or more parts of information from the parents. This case provides a stronger social learning capability, but the calculation is more complex. It is assumed that there are three parents: $x_{i}=\{x_{i1},\ldots ,x_{ij,}\ldots ,x_{ik},\ldots \}$, $y_{i}=\{y_{i1},\ldots ,y_{ij,}\ldots ,y_{ik},\ldots \}$, and $z_{i}=\{z_{i1},\ldots ,z_{ij,}\ldots ,z_{ik},\ldots \}$, and a possible fruit can be generated as shown in Equation (44).

(44) $$x_{i}^{\prime }(t)=\{x_{i1},\ldots ,y_{ij,}\ldots ,z_{ik},\ldots \}$$

Through the parthenogenesis and social learning of plant individuals, the fruiting step recovers the population size of the APC to the original value of S.

Step 5. End Justification:

After a lot of iterative computation, the edge AGVs should justify whether the computation should be stopped, and the optimal solution will be output to arrange the AGVs to complete production tasks and avoid collisions. The often-used justification conditions include the maximum number of iterations, ${Ite}_{max}$, the error threshold, $e_{th},$ or the maximum computation time. 

The best solution, $x_{i}^{\#}(t)$, at time t has the fitness $fit[x_{i}^{\#}\left(t\right)]$, as obtained through Equation (37). In the previous time, (t−1), the best solution, $x_{i}^{\#}(t-1)$, has the fitness $fit[x_{i}^{\#}\left(t-1\right)]$. The error between them can be given as

(45) $$error(t)=|fit\left[x_{i}^{\#}\left(t\right)\right]-fit[x_{i}^{\#}\left(t-1\right)]|$$

Therefore, an error threshold, $e_{th}$, can be predefined to justify whether the solution process should be stopped.

(46) $$\left\{\begin{array}{c} if\;error(t)\leq e_{th},then\;go\;to\;end.\\ else,\;then\;return\;to\;the\;seeding\;step. \end{array}\right.$$

If the AGVs detect that the error(t) between two iterations is not greater than the predefined threshold, $e_{th}$, then the calculation can be stopped, and the optimal solution, $x_{i}^{\#}\left(t\right)$, can be output. Otherwise, the fruits will be returned to the seeding step as the seeds in the next iterative computation, and this process will continue to repeat until the end justification is satisfied.

### 5.3. Algorithm Flow of the APC

**Figure 5:**
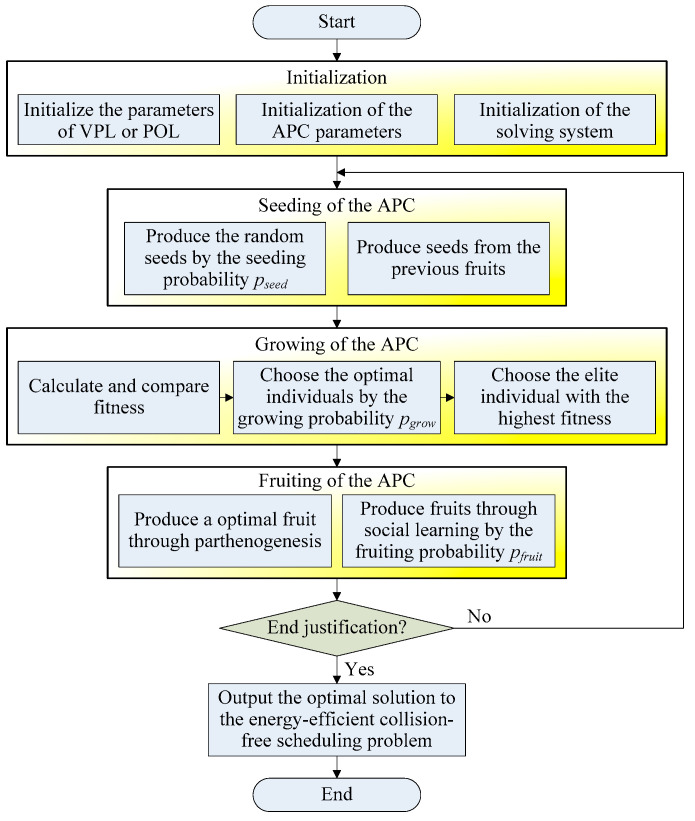
The APC algorithm flow for the energy-efficient collision-free machine/AGV scheduling problem.

The whole solution flow of the APC algorithm to solve the energy-efficient collision-free machine/AGV scheduling problem is shown in Figure 5, which is based on the swarm intelligence of edge AGVs. By continuously executing the seeding, growing, and fruiting operations, edge AGVs can, at last, search for the optimal solution to energy-efficient collision-free machine/AGV scheduling problems.

The algorithm flow contains five main steps and a large loop. The five main steps of the APC algorithm simulate the growth behavior of a natural plant community, and the whole calculation process employs simple operations and few parameters. Therefore, the APC algorithm has low hardware requirements and can be easily deployed in the embedded platforms of edge AGVs.

Based on the APC algorithm flow in Figure 5, the pseudo-codes for the VPL and POL are shown in Algorithms 1 and 2. Algorithm 1 describes the solving process for an energy-efficient collision-free machine/AGV scheduling problem in the virtual planning layer, where the APC individual is encoded by Equation (38). The fitness function in Equation (37), ${Obj}_{VPL}$ in Equation (12), and constraints in Equation (13) are employed for fitness calculation and comparison. The corresponding state transition can be referred to in Figure 2 and Figure 3.
**Algorithm 1:** Solving the energy-efficient collision-free machine/AGV scheduling problem in the virtual planning layer1:task state = new2:if AGV state = idle3:if assigned then task state = ready4:initialize the parameters of energy-efficient collision-free machine/AGV scheduling problem5:initialize the parameters of APC6:initialize the parameters of the solving system7:encode the APC individual by Equation (38)8:select fitness function by Equations (37) and (12)9:for ite = 1 to ${Ite}_{max}$
10: produce the random seeds by $p_{seed}$
11: produce seeds from the previous fruits12: calculate fitness by Equations (37) and (12)13: choose the best solutions by $p_{grow}$
14: choose the elite individual with the highest fitness15: choose a fruit through parthenogenesis16: produce fruits through social learning by $p_{fruit}$
17: end justification by $e_{th}$
18:end for19:output the optimal solution in the virtual planning layer20:if scheduled then task state = performing21:if interrupted then task state = waiting22:if completed then task state = exit
**Algorithm 2:** Solving the energy-efficient collision-free machine/AGV scheduling problem in the physical optimization layer1:if assigned then task state = optimization2:if machine/AGV state = idle3:input the best solution in the virtual planning layer4:initialize the parameters of energy-efficient collision-free machine/AGV scheduling problem5:initialize the parameters of APC6:initialize the parameters of the solving system7:encode the APC individual by Equation (39)8:select fitness function by Equations (37) and (28)9:for ite = 1 to ${Ite}_{max}$10: produce the random seeds by $p_{seed}$11: produce seeds from the previous fruits12: calculate fitness by Equations (37) and (28)13: choose the best solutions by $p_{grow}$14: choose the elite individual with the highest fitness15: produce a fruit through parthenogenesis16: produce fruits through social learning by $p_{fruit}$17: end justification by $e_{th}$18:end for19:output the best solution in the physical optimization layer20:if scheduled then task state = performing21:if assigned then machine/AGV state = loading22:if scheduled then machine/AGV state = processing/transportation23:if detected then machine/AGV state = obstacle avoidance24:if detected then machine/AGV state = obstacle avoidance25:if avoided then machine/AGV state = processing/transportation26:if completed then machine/AGV state = idle

After that, the best solution in the virtual planning layer will be output to the physical optimization layer for AGV assignment. Algorithm 2 depicts the solving process for energy-efficient collision-free machine/AGV scheduling problems in the physical optimization layer, where the APC individual is encoded by Equation (39). The fitness function in Equation (37), ${Obj}_{POL}$ in Equation (28), and the constraints in Equations (29)–(36) are chosen for fitness calculation and comparison. The corresponding state transition can be referred to in Figure 2 and Figure 3. 

## 6. Benchmark Dataset and Tests

In this section, a benchmark test conducted to verify the proposed VEI-based architecture is analyzed, including the benchmark settings, numerical experimental results, and comparative analysis.

### 6.1. Benchmark Dataset

To test and compare the performance of the energy-efficient collision-free machine/AGV scheduling problem, the benchmark data used in [11] were employed and modified here; the process was undertaken in a production workshop environment. According to the test requirements of our model, the benchmark test is modified as a case with 15 tasks, 15 machines, and 15 AGVs, which is more complex than the data in reference [11], which used 15 jobs, 8 machines, and 15 AGVs. The authors of [11] designed a unidirectional loop of the AGV route that had a total length of 900 m and an equal distance between the load/offload nodes of each consecutive machine of 100 m. 

The experimental setup and construction of the benchmark dataset were based on the production environment of reference [11] and simulated real-world production environments as much as possible. In real production environments, machines are usually neatly arranged in the factory building, taking into account the mobility and variability of AGVs. Our modified benchmark test employed a more complex production scenario with three transportation lines, and each transportation line has five machines. There are 15 AGVs to be allowed in, and they can enter or exit the node to balance the transportation load between AGVs. 

**Figure 6:**
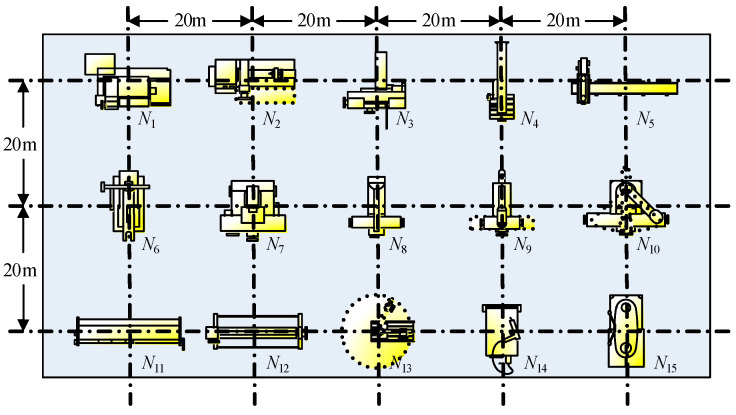
Benchmark roadmap in a production workshop.

By referring to the layout of the actual production system [11], Figure 6 illustrates the design of the benchmark test roadmap of the production workshop. There are 15 machines $N=\{N_{1},N_{2},\ldots ,N_{15}$}, with a 20 m interval between them to simplify the calculations and benchmark tests for international peers. Based on Figure 6, the transportation distances, $d_{ij}$, between consecutive machines are listed in Table 3.

The processing times, $t_{vi}^{proce}$, for 15 tasks $T=\left\{T_{1},T_{2},\ldots ,T_{15}\right\}$ and 15 machines $N=\{N_{1},N_{2},\ldots ,N_{15}$} are shown in Table 4, representing about twice the number of machines in the original benchmark data [11]. Without losing generality, this modification does not affect the effectiveness of the benchmark test and is more suitable for our complex production scenario. When compared with the transportation distances in Table 3 and the average speed of AGV (about 1 m/s), the processing times in Table 4 are greater, and the benchmark data are consistent with real-world cases [11]. The loads of cargo c are all set as $w_{c}$ = 10 Kg, and the maximum load capacity of AGV v is preset as $w_{v}^{max}=150$ kg. That is, an AGV can load 15 cargo at most each time. Then, the total load of all cargo in a task is $w_{\Sigma }$ = 150 kg.

We collected data on the driving speed of AGVs in real production environments, and the AGV speed is limited to between 0.4 m/s and 2 m/s, which is divided into five different speed levels. Table 5 gives the reference transportation power, $p_{v}^{driv}$, of the AGVs with different speed rates, which are adjusted from the travel power in the benchmark test data [11] to simulate AGV transportation. The energy consumption rate using a speed rate is calculated by dividing the no-load power by the speed values. Compared with the reference no-load power of the AGVs, the loaded power increases by a factor of 1.6, and the average power of an AGV to load/unload a cargo is 1.08 kW [11]. To sum up, an AGV consumes 3 Wh of energy for every loading/unloading operation of a task. 

Table 6 indicates the processing power, $p_{v}^{driv}$, consumed on the machines, which is adjusted from the setup power in the benchmark test data [11]. Therefore, the processing energy consumption can be calculated by multiplying the processing power values by the processing times. Since these data are introduced from the existing literature to fit the needs of benchmark tests and serve as a proxy to a real-world production system [11], this modification is more suitable for our benchmark tests. 

To simplify the experimental analysis, some assumptions are made.

**Assumption** **1.** *The differences between AGVs, machines, tasks, or cargo are not considered. Human interference, environmental factors, faults, and unexpected events are also not considered*.

**Assumption** **2.** *The movements of an AGV often include lifting/lowering the cargo, transporting the cargo from the AGV load platform to a machine or vice versa. Since these movements have a shorter duration, they are ignored here*.

**Assumption** **3.** *It is assumed that the battery of an AGV has sufficient power without considering transportation failures or charging caused by insufficient power. The assumption is reasonable because the battery of an AGV can complete multiple production tasks after being fully charged or does not require additional charging for each production task*.

**Assumption** **4.** *If the time spent by different AGVs on the same machine does not overlap, it can be considered that there is no collision, and the collision-oriented energy consumption can be neglected*.

### 6.2. Numerical Experimental Results

This section gives the numerical experimental results of the proposed APC algorithm. For the APC algorithm, the population size = 80, the seeding probability $p_{seed}=0.2$, the growing probability $p_{grow}=0.7$, the fruiting possibility $p_{fruit}=0.1$, and a cross factor $C_{fruit}=2$.

The benchmark tests are all based on an experimental platform using AMD Ryzen 3 4300U with Radeon Graphics 2.70 GHz CPU, 8.00 GB RAM, 64-bit Windows 10 operating system, and MATLAB R2018a simulation software. All algorithms had the maximum number of iterations set as ${Ite}_{max}=200$, and the maximum computation time for end justification = 0.5 h.

To test the proposed model, the multi-objective function in Equation (28) was employed for performance analysis, including the makespan, $t_{\Sigma }$, total energy consumption, $e_{\Sigma }$, the total collision time, $t_{\Sigma }^{colli}$, and the route balance index, $d_{Rv}^{balan}$. It can be seen that there are conflicts between these four objectives. 

**Figure 7:**
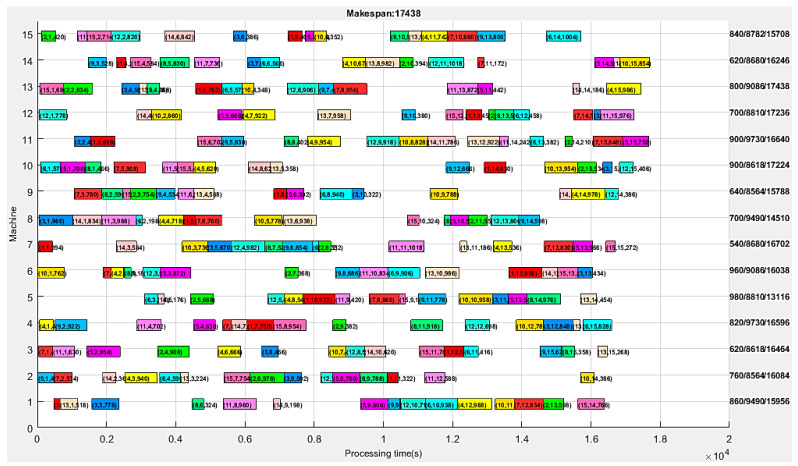
The processing time for energy-efficient collision-free AGV scheduling. (The same color is used for different processes of the same task, and different colors are randomly assigned to different tasks).

The processing time for the energy-efficient collision-free AGV scheduling is shown in Figure 7. The vertical axis represents the machine number, the horizontal axis represents the processing time, and the right side indicates the transportation time, processing time, and the makespan of each machine in fractional form. Figure 7 shows the machine scheduling results and Gantt chart under the minimum makespan, with different production tasks identified using different colors. Each progress bar on the Gantt chart is marked with three numbers enclosed in parentheses, representing the production task number, $T_{i}$, cargo number, $C_{c}$, and processing time, $t_{ci}^{proce}$, respectively. The makespan value in Figure 7 is $t_{\Sigma }=$ 17,438 s, and according to the corresponding processing time, the machine power consumption can be calculated to be $e_{i}=$ 41.334 kWh.

**Figure 8:**
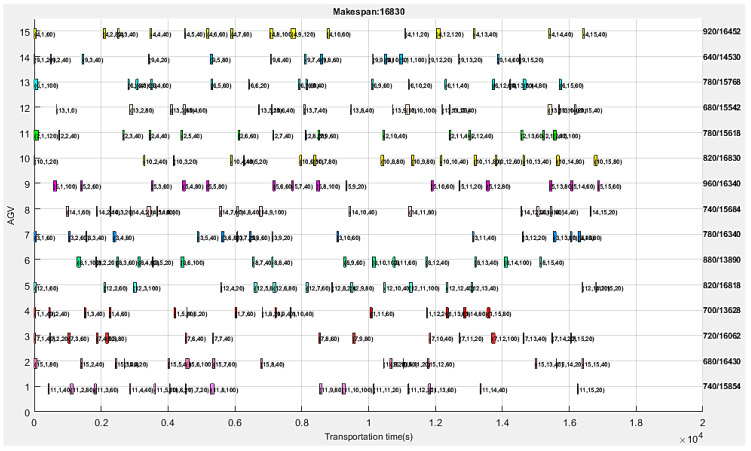
The transportation time for energy-efficient collision-free AGV scheduling. (The same color is used for different transportations of the same AGV, and different colors are randomly assigned to different AGVs).

The transportation time for energy-efficient collision-free AGV scheduling is given in Figure 8. The vertical axis indicates the AGV number, the horizontal axis represents the transportation time, and the right side indicates the transportation time and makespan of each AGV in fractional form. Figure 8 presents the AGV scheduling results and Gantt chart corresponding to the minimum makespan in Figure 7, with different production tasks filled with different colors. Each progress bar on the Gantt chart is marked with three numbers enclosed in parentheses, representing the production task number, cargo number, and transportation time, respectively. The longest travel time in Figure 8 is $t_{v}=$ 960 s, and the total AGV power consumption can be calculated to be $e_{v}=$ 0.618 kWh. Hence, the total energy consumption calculated by the proposed APC algorithm is $e_{\Sigma }=$ 41.952 kWh. The total collision time $t_{\Sigma }^{colli}$ = 0.00 s, so the proposed APC algorithm realizes the collision-free objective. The actual maximum transportation time in Figure 8 is $\max\left\{d_{Rv}\right\}=$ 960 s, and the actual minimum transportation time is $\min\left\{d_{Rv}\right\}=$ 640 s, so the degree of route balance can be obtained by calculating their deviation: $d_{Rv}^{balan}=\max\left\{d_{Rv}\right\}-\min\{d_{Rv}\}$ = 320 m.

In order to observe the experimental data more clearly, the Gantt data of processing times in Figure 7 are listed in Table 7, and the Gantt data of transportation times in Figure 8 are listed in Table 8. In Table 7, the first column is the machine number, corresponding to the vertical axis in Figure 7; The first line indicates the 15 processes on each machine, corresponding to the horizontal axis in Figure 7. Each data item in Table 7 corresponds one-to-one with Figure 7, and each item of data is a triplet enclosed in parentheses, representing the production task number, $T_{i}$, cargo number, $C_{c}$, and processing time, $t_{ci}^{proce}$, respectively. In Table 8, the first column is the AGV number, corresponding to the vertical axis in Figure 8; The first line indicates the 15 transports on each AGV, corresponding to the horizontal axis in Figure 8. Each data item in Table 8 corresponds one-to-one with Figure 8, and each item of data is a triplet enclosed in parentheses, representing the production task number, $T_{i}$, cargo number, $C_{c}$, and transportation time, $t_{v}$, respectively.

After more than 250 timed tests, the statistics of the optimal solutions of the benchmark test are shown in Table 9. As can be observed from Table 9, the minimum value of the makespan, $t_{\Sigma }$, shows a 12.88% improvement compared to the maximum value, and the minimum value of AGV energy consumption, $e_{\Sigma }$, shows a 0.09% improvement compared to the maximum value. This means that our APC algorithm is effective in improving energy efficiency. The total AGV energy consumption (kWh) is positively correlated with the maximum traveled distance (m) and the average AGV speed (m/s) in Table 9, but it is negatively correlated with the makespan. The total processing energy (kWh) of the machines is 63.07~66.86 times that of the AGVs. The total collision time is $t_{\Sigma }^{colli}$ = 0.00 s, which, again, verifies that the proposed APC algorithm realizes the collision-free objective.

**Figure 9:**
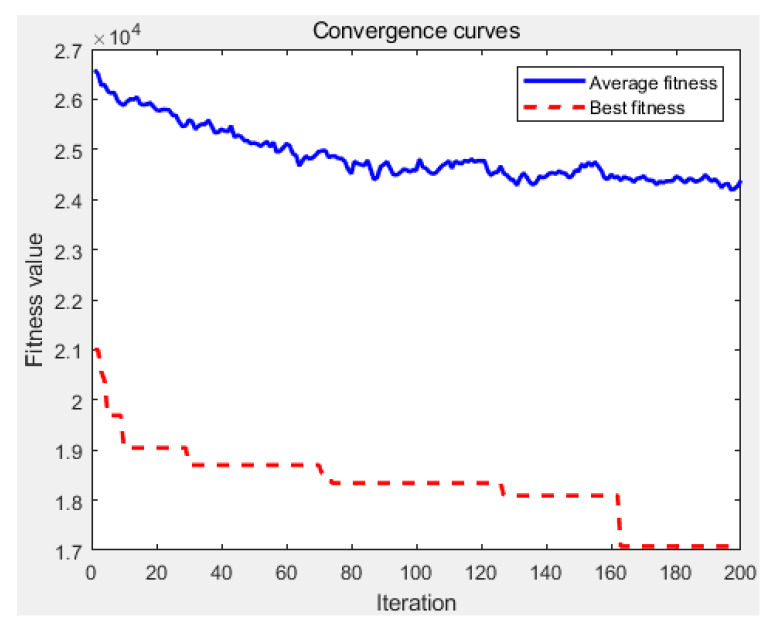
The convergence curves of the proposed APC algorithm.

The benchmark test results in Figure 7 and Figure 8 were obtained using the maximum iteration of 200. Figure 9 presents the convergence curves of the proposed APC algorithm. As we can see, the proposed method can obtain the optimal solution after about 163 iterations. This indicates that this algorithm has good convergence performance.

**Figure 10:**
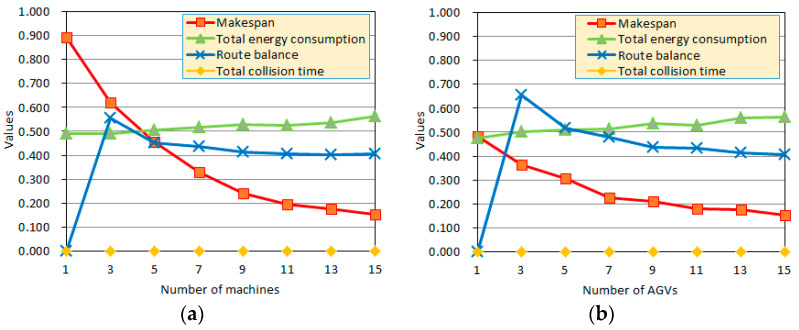
Comparison of parameter changes. ((**a**) The trends of the four main indicators with different machine numbers; (**b**) the trend of the four main indicators with different AGV numbers).

To further excavate the conflicting factors in energy-efficient collision-free AGV scheduling, Figure 10a,b show how the changing parameters affected four main indexes, i.e., the makespan, $t_{\Sigma }$, total energy consumption, $e_{\Sigma }$, the total collision time, $t_{\Sigma }^{colli}$, and the route balance index, $d_{Rv}^{balan}$. Due to the different dimensions of the four indicators, there is a significant difference in numerical values, so we have normalized them.

Figure 10a shows the trend of the four main indicators with different machine numbers. As we can see, the proposed APC algorithm realizes a collision-free objective of $t_{\Sigma }^{colli}$ = 0.00 s, and more machines are helpful in reducing the makespan, $t_{\Sigma }$; however, this increases the total energy consumption, $e_{\Sigma }$, because more machines need more AGVs and transportation routes. When the machine number is 7~15, the four indexes have no significant fluctuations since the cargo on each machine has a maximum of 10 nodes on each route. When the machine numbers decrease, it is helpful to reduce congestion and collision, but the makespan, $t_{\Sigma }$, and the route balance index, $d_{Rv}^{balan}$, increase since more time is required to wait for the idle machines.

Figure 10b shows the changes in the four main indicators in the solution results as the number of AGVs increases from small to large. It can be seen that the proposed APC algorithm realizes the collision-free objective of $t_{\Sigma }^{colli}$ = 0.00 s, and more AGVs are helpful in decreasing the makespan, $t_{\Sigma }$, but this increases the total energy consumption, $e_{\Sigma }$, because more AGVs produce more transportation routes. When the number of AGVs decreases, the traffic density decreases, reflecting less congestion and fewer collisions, but the route balance index, $d_{Rv}^{balan}$, increases for longer routes. If the AGV number is small, the makespan will be greater, which is negative in terms of production efficiency.

When combined with Figure 10a,b, the machine energy consumption and AGV energy consumption are greater than that of collision avoidance, so it is important to maintain production efficiency with a low makespan, $t_{\Sigma }$. From Figure 10a,b, reducing the number of machines or AGVs can lead to an increase in the makespan values, $t_{\Sigma }$, and a decrease in production efficiency. However, the increase in the makespan value, $t_{\Sigma }$, caused by reducing the number of machines is greater than that of reducing the AGVs. The average speed with respect to the minimum makespan can be used to choose an economical speed and load. In engineering applications, higher productivity requires more machines, AGVs, and more energy consumption to load/unload more cargo at a higher speed.

### 6.3. Comparative Analysis

This section compares the performance of the proposed APC algorithm and some state-of-the-art methods for different numbers of AGVs, including the proposed APC, GA [9,17], ACO [14,27,34], PSO [29,30], and DQN [25,31]. All these SOTA methods are based on the benchmark datasets and the multi-objective function in Equation (28). 

The GA sets the population size to m = 80, chromosome length to Lind = 20, crossover probability to px = 0.7, and mutation probability to pm = 0.01. The parameters of ACO are set as follows: the pheromone importance = 1.0, the importance of heuristic factors = 5.0, the pheromone volatilization factor = 0.1, and m = 80 ants. The PSO sets the population size to m = 80, the location limitation to 0.5, the speed limitation to [−0.5, 0.5], the self-learning factor to c1 = 1.5, and the social learning factor to c2 = 1.5. DQN sets the following parameters: experience pool capacity = 500, learning rate 2 × 10^−3^, discount factor = 0.9, greed coefficient = 0.9, the update frequency of the parameters of the target network = 200, batch size = 32, and the number of hidden layer neurons = 128.

Four main metrics are used to compare performance, i.e., the makespan $t_{\Sigma }$, total energy consumption, $e_{\Sigma }$, the route balance index, $d_{Rv}^{balan}$, and the solution time. Because the problem scale and the number of AGVs affect the results of the metrics, the calculated results of the objective function are normalized in the range of 0 to 1. The solution time is provided in seconds. Two cases are compared here, including different machine numbers and different AGV numbers. The number of machines increases from $N_{max}=1,3,5,\ldots$ to $N_{max}=15$, and the number of AGVs increases from $V_{max}=1,3,5,\ldots$ to $V_{max}=15$. Each algorithm runs for the same number of iterations and the same amount of computational time. Moreover, the optimal results for two scenarios in Table 10 and Table 11 are highlighted in bold font for comparison.

Table 10 provides a comparison of the results of benchmark tests to measure the four metrics of five heuristic techniques employed using different machine numbers. The first column lists the techniques: APC, GA, ACO, PSO, and DQN; each uses a basic version and the main preset parameters, as in Section 6 A. The first line of Table 10 indicates the number of machines, and there are nine cases in total, including the mean value. As we can see from the heuristic dispatching information, more machines are helpful in reducing the makespan for all algorithms, but this increases the energy consumption. It can be observed that the makespan, $t_{\Sigma }$, and total energy consumption, $e_{\Sigma }$, improve differently depending on the techniques used. However, the benchmark test verifies the proposed APC technique and makes it easier to search for the best solution in a short time; the best mean values for the makespan and energy consumption are shown in bold type. Compared with the main metrics of other algorithms, the proposed APC algorithm can improve the makespan by up to 8.61% and improve the energy consumption by up to 2.63% under the premise of no collision.

Table 11 provides the comparison results of the benchmark tests to measure the four metrics of five heuristic techniques employed using different AGV numbers. The APC, GA, ACO, PSO, and DQN adopt the same parameters as above, and the first line of Table 9 indicates the numbers of AGVs. The experimental results in Table 11 indicate that different algorithms can optimize metrics to varying degrees, but the proposed APC algorithm can obtain the best mean values for each metric. Compared with the main metrics of other algorithms, the proposed APC algorithm can improve the makespan by up to 8.78% and energy consumption by up to 2.42% under the premise of no collision. In terms of solution time, APC is better than DQN, though the DQN algorithm can search for optimal solutions but requires more computational time. Therefore, the APC algorithm has fast convergence, global search, and local optimum avoidance. 

As for the results in Table 10 and Table 11, the most effective improvements can be seen for APC, followed by DQN, and then GA. It is clear that the proposed APC algorithm makes obtaining optimal solutions easier than most STOA methods. The high effectiveness of APC in terms of distributed computation is suitable for the vehicle edge intelligence architecture and the energy-efficient collision-free machine/AGV scheduling problem. To obtain a lower makespan without collision, AGVs have to be arranged to exploit the available routes and perform more loaded/unloaded operations. In addition, when the number of AGVs increases, the proposed APC algorithm can obtain a stronger edge computing capability.

## 7. Conclusions

This paper proposes a VEI-based architecture to solve the energy-efficient collision-free machine/AGV scheduling problem. The proposed system includes a virtual planning layer and a physical optimization layer. The virtual planning layer assigns the production task to multiple AGVs according to uneven cargo, and the physical optimization layer optimizes energy-efficient obstacle avoidance. Both layers employ a heuristic artificial plant community algorithm to plan and optimize the makespan and energy consumption. The proposed method makes full use of existing production systems and exploits the potential of edge computing. The application of vehicle edge intelligence to a machine/AGV system is a new and urgent requirement that merges artificial intelligence and edge computing into a more efficient operational system.

This paper has four main contributions. First, the production system is based on a vehicle edge intelligence architecture and makes the best of the edge computing capabilities of multiple AGVs. Second, the multi-objective functions in the VPL and POL consider production efficiency, collision avoidance, and energy efficiency, and the hierarchical scheduling method improves the operational efficiency of a machine/AGV system. Third, an artificial plant community algorithm was developed to solve the energy-efficient collision-free machine/AGV scheduling problem. The algorithm is simple in terms of operation and the parameters used, low in hardware platform requirements, and suitable for deployment on edge computing devices. Finally, a benchmark dataset was introduced and modified to test the proposed method and compare it with state-of-the-art algorithms; our modified benchmark dataset can be employed for other similar tests.

The shortcomings of this study include several aspects: First, the collision avoidance and energy optimization problem in the machine/AGV system is affected by many complex factors; however, to simplify the analysis, some factors were overlooked or not taken into consideration. Second, the application of vehicle edge intelligence in the production system is very limited, so there are few successful cases for reference. Hence, our benchmark data need to be verified by other researchers. Third, our proposed algorithm may not be perfect and requires more improvement and tests, such as using more metrics to test scalability to larger systems.

Future research work should focus on the following directions: First, more factors should be considered in the energy-efficient collision-free machine/AGV scheduling problem. Second, more task assignments and more AGV types should be surveyed in vehicle edge intelligence. Third, the VEI method can be further improved and tested in larger production systems.

## Figures and Tables

**Table 1:** Definition and comparison of VEI.

	The Virtual Planning Layer	The Physical Optimization Layer
Level	Top layer	Bottom layer
Definition	Virtual entity	Physical entity
Role	Edge servers	Edge AGVs
Attribute	Fixed	Mobile
Function	Pre-arrange	Real time
Architecture	Integrated	Distributed
Computational ability	Server level	Embedded platforms
Storage capacity	Server level	Embedded platforms

**Table 2:** Notations and explanations.

Notations	Explanation
T	A production task
t	Time
Obj	The objective function
Λ	An arc set
N	A set of machines
E	A set of edges
C	A set of cargo
V	A set of AGVs
i,j	The node number
v	The AGV number
c	The cargo number
$T_{max}$	Total number of tasks
$N_{max}$	Total number of machines
$C_{max}$	Total number of cargoes
$V_{max}$	Total number of AGVs
$e_{ij}$	An edge between the nodes i and j
$d_{ij}$	The distance between the two nodes i and j
$w_{\Sigma }$	The total load of all cargo in a task
$w_{c}$	The load of cargo c
$w_{v}$	The load of AGV v
$w_{v}^{max}$	The maximum load capacity of AGV v
$L_{v}$	The load factor of AGV v
$L_{v}^{max}$	The maximum load factor of AGV v
$L_{v}^{min}$	The minimum load factor of AGV v
$R_{v}$	The route of AGV v
$d_{Rv}$	The total distance of route $R_{v}$
$d_{Rv}^{balan}$	The route balance index of AGV v
$\lambda_{vc}$	A selection bit of AGV v, ∈{0,1}. =1 if cargo c is on AGV v; otherwise, =0.
$\lambda_{vij}$	A selection bit of route $R_{v}$, ∈{0,1}. =1 if $e_{ij}$ is on route $R_{v}$; otherwise, =0.
a	The start node of route $R_{v}$
b	The end node of route $R_{v}$
$s_{v}$	The driving speed of AGV v
$s_{v}^{max}$	The maximum driving speed of AGV v
$B_{v}^{max}$	The battery capacity of AGV v
$e_{\Sigma }$	The total energy consumption of a task
$e_{v}$	The total energy consumption of AGV v
$e_{i}$	The total energy consumption of machine i
$e_{vij}$	The energy consumption of AGV v on edge $e_{ij}$
$e_{ci}$	The energy consumption of cargo c on node i
$p_{v}^{driv}$	The driving power of AGV v
$p_{v}^{stop}$	The stop power of AGV v
$p_{v}^{turn}$	The turning power of AGV v
$p_{v}^{acce}$	The acceleration power of AGV v
$p_{ci}^{proce}$	The processing power when cargo c is on node i
$F_{v}^{driv}$	The driving force of AGV v
$F_{v}^{stop}$	The stop force of AGV v
$F_{v}^{turn}$	The turning force of AGV v
$F_{v}^{acce}$	The acceleration force of AGV v
$t_{vij}$	The unit time window of AGV v on edge $e_{ij}$
$t_{\Sigma }$	The total time cost (makespan) of a task
$t_{v}$	The longest travel time of AGVs
$t_{i}$	The total time cost of machine i
$t_{\Sigma }^{colli}$	The total collision time on all nodes
$t_{vi}^{enter}$	The time when AGV v enters node i
$t_{vi}^{leave}$	The time when AGV v leaves the current node i
$t_{ci}^{proce}$	The processing time when cargo c is on node i
$t_{vi}^{colli}$	The collision time on node i
$t_{vij}^{stop}$	The stop time of AGV v on edge $e_{ij}$
$t_{vij}^{turn}$	The turning time of AGV v on edge $e_{ij}$
$t_{vij}^{acce}$	The acceleration time of AGV v on edge $e_{ij}$

**Table 3:** The transportation distance (m) between consecutive nodes.

Machines	$N_{1}$	$N_{2}$	$N_{3}$	$N_{4}$	$N_{5}$	$N_{6}$	$N_{7}$	$N_{8}$	$N_{9}$	$N_{10}$	$N_{11}$	$N_{12}$	$N_{13}$	$N_{14}$	$N_{15}$
$N_{1}$	0	20	40	60	80	20	40	60	80	100	40	60	80	100	120
$N_{2}$	20	0	20	40	60	40	20	40	60	80	60	40	60	80	100
$N_{3}$	40	20	0	20	40	60	40	20	40	60	80	60	40	60	80
$N_{4}$	60	40	20	0	20	80	60	40	20	40	100	80	60	40	60
$N_{5}$	80	60	40	20	0	100	80	60	40	20	120	100	80	60	40
$N_{6}$	20	40	60	80	100	0	20	40	60	80	20	40	60	80	100
$N_{7}$	40	20	40	60	80	20	0	20	40	60	40	20	40	60	80
$N_{8}$	60	40	20	40	60	40	20	0	20	40	60	40	20	40	60
$N_{9}$	80	60	40	20	40	60	40	20	0	20	80	60	40	20	40
$N_{10}$	100	80	60	40	20	80	60	40	20	0	100	80	60	40	20
$N_{11}$	40	60	80	100	120	20	40	60	80	100	0	20	40	60	80
$N_{12}$	60	40	60	80	100	40	20	40	60	80	20	0	20	40	60
$N_{13}$	80	60	40	60	80	60	40	20	40	60	40	20	0	20	40
$N_{14}$	100	80	60	40	60	80	60	40	20	40	60	40	20	0	20
$N_{15}$	120	100	80	60	40	100	80	60	40	20	80	60	40	20	0

**Table 4:** The processing times (s) of the production tasks.

Cargo	$T_{1}$	$T_{2}$	$T_{3}$	$T_{4}$	$T_{5}$	$T_{6}$	$T_{7}$	$T_{8}$	$T_{9}$	$T_{10}$	$T_{11}$	$T_{12}$	$T_{13}$	$T_{14}$	$T_{15}$
$C_{1}$	180	598	778	988	806	938	834	324	332	552	960	718	518	198	766
$C_{2}$	322	978	502	940	780	596	534	788	456	386	588	348	224	368	754
$C_{3}$	570	908	466	666	954	416	406	358	622	492	630	534	268	620	708
$C_{4}$	750	382	848	402	630	828	242	918	922	786	702	698	210	458	954
$C_{5}$	922	668	458	540	548	380	860	976	778	958	420	490	454	176	182
$C_{6}$	956	368	434	348	872	906	228	186	686	762	834	500	986	442	578
$C_{7}$	394	352	670	536	566	172	830	528	854	736	1018	982	186	584	272
$C_{8}$	332	552	960	718	518	198	766	180	598	778	988	806	938	834	324
$C_{9}$	368	754	322	978	502	940	780	596	534	788	456	386	588	348	224
$C_{10}$	630	534	268	620	708	570	908	466	666	954	416	406	358	622	492
$C_{11}$	698	210	458	954	750	382	848	402	630	828	242	918	922	786	702
$C_{12}$	454	176	182	922	668	458	540	548	380	860	976	778	958	420	490
$C_{13}$	762	834	500	986	442	578	956	368	434	348	872	906	228	186	686
$C_{14}$	272	394	352	670	536	566	172	830	528	854	736	1018	982	186	584
$C_{15}$	406	420	386	742	254	1004	860	526	806	352	270	820	380	842	714

**Table 5:** The power consumption for different speed rates.

Rate	1	2	3	4	5
Speed (m/s)	0.4	0.5	0.67	1.0	2.0
Power consumption (W)	60.4	82.8	119.2	191.2	405.8
The load factor, $L_{v}$	1.0	1.0	1.0	0.9	0.8

**Table 6:** The processing power (kW) for each machine.

$N_{1}$	$N_{2}$	$N_{3}$	$N_{4}$	$N_{5}$	$N_{6}$	$N_{7}$	$N_{8}$	$N_{9}$	$N_{10}$	$N_{11}$	$N_{12}$	$N_{13}$	$N_{14}$	$N_{15}$
1.22	1.22	1.03	1.03	1.14	1.14	1.01	1.01	1.12	1.12	1.21	1.21	1.10	1.10	0.90

**Table 7:** Gantt data of the processing time in Figure 7.

	Process	1	2	3	4	5	6	7	8	9	10	11	12	13	14	15
Machine																
15		(2, 1, 420)	(11, 2, 270)	(15, 2, 714)	(12, 2, 820)	(14, 6, 842)	(3, 6, 386)	(1, 9, 406)	(5, 7, 254)	(10, 6, 352)	(8, 10, 526)	(13, 9, 380)	(4, 11, 742)	(7, 10, 860)	(9, 13, 806)	(6, 14, 1004)
14		(9, 3, 528)	(1, 4, 272)	(15, 4, 584)	(8, 5, 830)	(11, 7, 736)	(3, 7, 352)	(6, 6, 566)	(4, 10, 670)	(13, 8, 982)	(2, 10, 394)	(12, 11, 1018)	(7, 11, 172)	(5, 14, 536)	(14, 15, 186)	(10, 15, 854)
13		(15, 1, 686)	(2, 2, 834)	(3, 4, 500)	(13, 2, 228)	(8, 4, 368)	(1, 6, 762)	(6, 5, 578)	(10, 4, 348)	(12, 6, 906)	(9, 7, 434)	(7, 8, 956)	(11, 13, 872)	(5, 11, 442)	(14, 14, 186)	(4, 15, 986)
12		(12, 1, 778)	(14, 4, 420)	(10, 2, 860)	(5, 5, 668)	(4, 7, 922)	(13, 7, 958)	(9, 10, 380)	(15, 12, 490)	(1, 13, 454)	(2, 12, 176)	(8, 13, 548)	(6, 12, 458)	(7, 14, 540)	(3, 14, 182)	(11, 15, 976)
11		(3, 2, 458)	(1, 3, 698)	(15, 6, 702)	(9, 5, 630)	(8, 8, 402)	(4, 9, 954)	(12, 9, 918)	(10, 8, 828)	(14, 11, 786)	(13, 12, 922)	(11, 14, 242)	(6, 13, 382)	(2, 14, 210)	(7, 15, 848)	(5, 15, 750)
10		(6, 1, 570)	(5, 1, 708)	(8, 1, 466)	(7, 5, 908)	(11, 5, 416)	(15, 5, 492)	(4, 5, 620)	(14, 8, 622)	(13, 5, 358)	(9, 12, 666)	(1, 14, 630)	(10, 13, 954)	(2, 15, 534)	(3, 15, 268)	(12, 15, 406)
9		(7, 3, 780)	(8, 2, 596)	(15, 3, 224)	(2, 3, 754)	(9, 4, 534)	(11, 6, 456)	(13, 4, 588)	(1, 8, 368)	(5, 6, 502)	(6, 8, 940)	(3, 10, 322)	(10, 9, 788)	(14, 13, 348)	(4, 14, 978)	(12, 14, 386)
8		(3, 1, 960)	(14, 1, 834)	(11, 3, 988)	(6, 2, 198)	(4, 4, 718)	(1, 5, 332)	(7, 6, 766)	(10, 5, 778)	(13, 6, 938)	(15, 10, 324)	(8, 12, 180)	(5, 10, 518)	(2, 11, 552)	(12, 13, 806)	(9, 14, 598)
7		(1, 1, 394)	(14, 3, 584)	(10, 3, 736)	(3, 5, 670)	(12, 4, 982)	(8, 7, 528)	(9, 6, 854)	(6, 7, 172)	(2, 8, 352)	(11, 11, 1018)	(13, 11, 186)	(4, 13, 536)	(7, 13, 830)	(5, 13, 566)	(15, 15, 272)
6		(10, 1, 762)	(7, 4, 228)	(4, 2, 348)	(8, 3, 186)	(12, 3, 500)	(5, 3, 872)	(2, 7, 368)	(9, 8, 686)	(11, 10, 834)	(6, 9, 906)	(13, 10, 986)	(1, 15, 956)	(14, 12, 442)	(15, 13, 578)	(3, 13, 434)
5		(6, 3, 380)	(14, 5, 176)	(2, 5, 668)	(12, 5, 490)	(4, 8, 540)	(1, 10, 922)	(11, 9, 420)	(7, 9, 860)	(15, 9, 182)	(9, 11, 778)	(10, 10, 958)	(3, 11, 458)	(5, 12, 548)	(8, 14, 976)	(13, 14, 454)
4		(4, 1, 402)	(9, 2, 922)	(11, 4, 702)	(5, 4, 630)	(7, 7, 242)	(14, 7, 458)	(1, 7, 750)	(15, 8, 954)	(2, 9, 382)	(8, 11, 918)	(12, 12, 698)	(10, 12, 786)	(3, 12, 848)	(13, 13, 210)	(6, 15, 828)
3		(7, 1, 406)	(11, 1, 630)	(5, 2, 954)	(2, 4, 908)	(4, 6, 666)	(3, 8, 466)	(10, 7, 492)	(12, 8, 534)	(14, 10, 620)	(15, 11, 708)	(1, 12, 570)	(6, 11, 416)	(9, 15, 622)	(8, 15, 358)	(13, 15, 268)
2		(9, 1, 456)	(7, 2, 534)	(14, 2, 368)	(4, 3, 940)	(6, 4, 596)	(13, 3, 224)	(15, 7, 754)	(2, 6, 978)	(3, 9, 502)	(12, 7, 348)	(5, 8, 780)	(8, 9, 788)	(1, 11, 322)	(11, 12, 588)	(10, 14, 386)
1		(1, 2, 180)	(13, 1, 518)	(3, 3, 778)	(8, 6, 324)	(11, 8, 960)	(14, 9, 198)	(5, 9, 806)	(9, 9, 332)	(12, 10, 718)	(6, 10, 938)	(4, 12, 988)	(10, 11, 552)	(7, 12, 834)	(2, 13, 598)	(15, 14, 766)

**Table 8:** Gantt data of the transportation time in Figure 8.

	Transportation	1	2	3	4	5	6	7	8	9	10	11	12	13	14	15
AGV																
15		(4, 1, 60)	(4, 2, 80)	(4, 3, 40)	(4, 4, 40)	(4, 5, 40)	(4, 6, 60)	(4, 7, 60)	(4, 8, 100)	(4, 9, 120)	(4, 10, 60)	(4, 11, 20)	(4, 12, 120)	(4, 13, 40)	(4, 14, 40)	(4, 15, 40)
14		(9, 1, 20)	(9, 2, 40)	(9, 3, 40)	(9, 4, 20)	(9, 5, 80)	(9, 6, 40)	(9, 7, 40)	(9, 8, 60)	(9, 9, 20)	(9, 10, 60)	(9, 11, 100)	(9, 12, 20)	(9, 13, 20)	(9, 14, 60)	(9, 15, 20)
13		(6, 1, 100)	(6, 2, 40)	(6, 3, 60)	(6, 4, 60)	(6, 5, 60)	(6, 6, 20)	(6, 7, 60)	(6, 8, 40)	(6, 9, 60)	(6, 10, 20)	(6, 11, 40)	(6, 12, 60)	(6, 13, 20)	(6, 14, 80)	(6, 15, 60)
12		(13, 1, 0)	(13, 2, 80)	(13, 3, 60)	(13, 4, 60)	(13, 5, 20)	(13, 6, 40)	(13, 7, 40)	(13, 8, 40)	(13, 9, 20)	(13, 10, 100)	(13, 11, 20)	(13, 12, 40)	(13, 13, 100)	(13, 14, 20)	(13, 15, 40)
11		(2, 1, 120)	(2, 2, 40)	(2, 3, 40)	(2, 4, 40)	(2, 5, 40)	(2, 6, 60)	(2, 7, 40)	(2, 8, 20)	(2, 9, 60)	(2, 10, 40)	(2, 11, 40)	(2, 12, 40)	(2, 13, 60)	(2, 14, 40)	(2, 15, 100)
10		(10, 1, 20)	(10, 2, 40)	(10, 3, 20)	(10, 4, 40)	(10, 5, 20)	(10, 6, 60)	(10, 7, 80)	(10, 8, 80)	(10, 9, 80)	(10, 10, 40)	(10, 11, 80)	(10, 12, 60)	(10, 13, 40)	(10, 14, 80)	(10, 15, 80)
9		(5, 1, 100)	(5, 2, 60)	(5, 3, 60)	(5, 4, 80)	(5, 5, 80)	(5, 6, 60)	(5, 7, 40)	(5, 8, 100)	(5, 9, 20)	(5, 10, 60)	(5, 11, 20)	(5, 12, 80)	(5, 13, 80)	(5, 14, 60)	(5, 15, 60)
8		(14, 1, 60)	(14, 2, 40)	(14, 3, 20)	(14, 4, 20)	(14, 5, 100)	(14, 6, 40)	(14, 7, 60)	(14, 8, 40)	(14, 9, 100)	(14, 10, 40)	(14, 11, 80)	(14, 12, 20)	(14, 13, 60)	(14, 14, 40)	(14, 15, 20)
7		(3, 1, 60)	(3, 2, 60)	(3, 3, 40)	(3, 4, 80)	(3, 5, 40)	(3, 6, 80)	(3, 7, 20)	(3, 8, 60)	(3, 9, 20)	(3, 10, 60)	(3, 11, 40)	(3, 12, 20)	(3, 13, 80)	(3, 14, 40)	(3, 15, 80)
6		(8, 1, 100)	(8, 2, 20)	(8, 3, 60)	(8, 4, 60)	(8, 5, 20)	(8, 6, 100)	(8, 7, 40)	(8, 8, 40)	(8, 9, 60)	(8, 10, 100)	(8, 11, 60)	(8, 12, 40)	(8, 13, 40)	(8, 14, 100)	(8, 15, 40)
5		(12, 1, 60)	(12, 2, 60)	(12, 3, 100)	(12, 4, 20)	(12, 5, 80)	(12, 6, 80)	(12, 7, 60)	(12, 8, 20)	(12, 9, 80)	(12, 10, 40)	(12, 11, 100)	(12, 12, 40)	(12, 13, 40)	(12, 14, 20)	(12, 15, 20)
4		(1, 1, 40)	(1, 2, 40)	(1, 3, 40)	(1, 4, 60)	(1, 5, 40)	(1, 6, 20)	(1, 7, 60)	(1, 8, 20)	(1, 9, 40)	(1, 10, 40)	(1, 11, 60)	(1, 12, 20)	(1, 13, 60)	(1, 14, 80)	(1, 15, 80)
3		(7, 1, 40)	(7, 2, 20)	(7, 3, 60)	(7, 4, 60)	(7, 5, 80)	(7, 6, 40)	(7, 7, 40)	(7, 8, 60)	(7, 9, 80)	(7, 10, 40)	(7, 11, 20)	(7, 12, 100)	(7, 13, 40)	(7, 14, 20)	(7, 15, 20)
2		(15, 1, 80)	(15, 2, 40)	(15, 3, 40)	(15, 4, 20)	(15, 5, 40)	(15, 6, 100)	(15, 7, 60)	(15, 8, 40)	(15, 9, 20)	(15, 10, 60)	(15, 11, 20)	(15, 12, 60)	(15, 13, 40)	(15, 14, 20)	(15, 15, 40)
1		(11, 1, 40)	(11, 2, 80)	(11, 3, 60)	(11, 4, 40)	(11, 5, 40)	(11, 6, 20)	(11, 7, 20)	(11, 8, 100)	(11, 9, 80)	(11, 10, 100)	(11, 11, 20)	(11, 12, 20)	(11, 13, 60)	(11, 14, 40)	(11, 15, 20)

**Table 9:** General statistics of the benchmark data.

Indicator	Solutions		
	Minimum	Average	Maximum
The makespan $t_{\Sigma }$ of a task (s)	17,438	18,455.5	20,016
The total energy consumption $e_{\Sigma }$ of a task (kWh)	41.952	41.975	41.989
The total collision time $t_{\Sigma }^{colli}$ on all nodes (s)	0.00	0.00	0.00
The route balance index $d_{Rv}^{balan}$ of AGV v (m)	280	320	360
The longest travel time $t_{v}$ of AGV v (s)	920	965	1000
Total processing energy (kWh)	41.334	41.334	41.334
Total AGV energy consumption (kWh)	0.618	0.642	0.655
Total traveled distance (m)	11,640	12,085	12,340
The lowest AGV speed (m/s)	1.0	1.0	1.0

**Table 10:** Algorithm comparison with different machine numbers.

Method		Number of Machines								Mean
		$N_{max}=1$	$N_{max}=3$	$N_{max}=5$	$N_{max}=7$	$N_{max}=9$	$N_{max}=11$	$N_{max}=13$	$N_{max}=15$	
APC	Makespan $t_{\Sigma }$	**0.893**	0.622	**0.455**	**0.329**	0.241	0.197	**0.176**	**0.154**	**0.383**
	Total energy consumption $e_{\Sigma }$	**0.489**	**0.491**	0.506	0.516	**0.528**	0.523	0.537	**0.563**	**0.519**
	Total collision time $t_{\Sigma }^{colli}$	**0.000**	**0.000**	**0.000**	**0.000**	**0.000**	**0.000**	**0.000**	**0.000**	**0.000**
	Route balance index $d_{Rv}^{balan}$	**0.000**	0.557	**0.453**	0.438	0.415	**0.405**	**0.402**	**0.407**	**0.385**
	Solution time (s)	412	405	428	433	447	446	459	461	436.375
GA	Makespan $t_{\Sigma }$	0.922	0.639	0.504	0.353	**0.240**	0.245	0.212	0.172	0.411
	Total energy consumption $e_{\Sigma }$	0.494	0.503	0.519	0.532	0.536	**0.521**	0.547	0.575	0.528
	Total collision time $t_{\Sigma }^{colli}$	**0.000**	**0.000**	**0.000**	**0.000**	**0.000**	**0.000**	**0.000**	**0.000**	**0.000**
	Route balance index $d_{Rv}^{balan}$	**0.000**	0.568	0.457	0.447	**0.412**	0.421	0.409	0.413	0.391
	Solution time (s)	401	**396**	438	436	443	454	450	**459**	434.625
ACO	Makespan $t_{\Sigma }$	0.928	0.657	0.472	0.366	0.260	0.253	0.209	0.166	0.414
	Total energy consumption $e_{\Sigma }$	0.498	0.507	0.522	0.539	0.541	0.546	0.544	0.568	0.533
	Total collision time $t_{\Sigma }^{colli}$	**0.000**	**0.000**	**0.000**	**0.000**	**0.000**	**0.000**	**0.000**	**0.000**	**0.000**
	Route balance index $d_{Rv}^{balan}$	**0.000**	0.563	0.466	**0.434**	0.426	0.418	0.411	0.422	0.393
	Solution time (s)	**396**	414	413	431	**439**	**422**	445	475	**429.375**
PSO	Makespan $t_{\Sigma }$	0.934	0.660	0.491	0.372	0.274	0.228	0.223	0.174	0.420
	Total energy consumption $e_{\Sigma }$	0.505	0.496	0.511	0.530	0.534	0.539	0.552	0.580	0.531
	Total collision time $t_{\Sigma }^{colli}$	**0.000**	**0.000**	**0.000**	**0.000**	**0.000**	**0.000**	**0.000**	**0.000**	**0.000**
	Route balance index $d_{Rv}^{balan}$	**0.000**	**0.549**	0.475	0.460	0.423	0.412	0.415	0.420	0.394
	Solution time (s)	419	398	**411**	**425**	442	453	**447**	472	433.375
DQN	Makespan $t_{\Sigma }$	0.916	**0.620**	0.458	0.331	0.256	**0.195**	0.189	0.160	0.391
	Total energy consumption $e_{\Sigma }$	0.493	0.518	**0.497**	**0.514**	0.529	0.532	**0.535**	0.564	0.523
	Total collision time $t_{\Sigma }^{colli}$	**0.000**	**0.000**	**0.000**	**0.000**	**0.000**	**0.000**	**0.000**	**0.000**	**0.000**
	Route balance index $d_{Rv}^{balan}$	**0.000**	0.559	0.454	0.449	0.418	0.406	0.405	**0.407**	0.387
	Solution time (s)	1845	1967	2084	2008	2016	2120	2171	2163	2046.75

**Table 11:** Algorithm comparison with different AGV numbers.

Method		Number of AGVs								Mean
		$V_{max}=1$	$V_{max}=3$	$V_{max}=5$	$V_{max}=7$	$V_{max}=9$	$V_{max}=11$	$V_{max}=13$	$V_{max}=15$	
APC	Makespan $t_{\Sigma }$	0.481	**0.364**	**0.307**	0.226	**0.212**	**0.179**	0.176	**0.154**	**0.262**
	Total energy consumption $e_{\Sigma }$	**0.475**	0.502	**0.508**	**0.514**	0.537	0.530	0.558	**0.563**	**0.523**
	Total collision time $t_{\Sigma }^{colli}$	**0.000**	**0.000**	**0.000**	**0.000**	**0.000**	**0.000**	**0.000**	**0.000**	**0.000**
	Route balance index $d_{Rv}^{balan}$	**0.000**	**0.653**	0.518	0.479	**0.436**	0.433	**0.415**	**0.407**	**0.418**
	Solution time (s)	410	415	426	427	435	442	454	461	433.750
GA	Makespan $t_{\Sigma }$	0.534	0.387	0.315	0.258	0.241	0.196	**0.173**	0.172	0.285
	Total energy consumption $e_{\Sigma }$	0.492	**0.495**	0.514	0.519	0.523	0.547	**0.548**	0.575	0.527
	Total collision time $t_{\Sigma }^{colli}$	**0.000**	**0.000**	**0.000**	**0.000**	**0.000**	**0.000**	**0.000**	**0.000**	**0.000**
	Route balance index $d_{Rv}^{balan}$	**0.000**	0.664	0.545	0.512	0.476	0.441	0.437	0.413	0.436
	Solution time (s)	416	411	**413**	**425**	434	458	451	**459**	**433.375**
ACO	Makespan $t_{\Sigma }$	0.523	0.389	0.331	0.247	0.240	0.195	0.184	0.166	0.284
	Total energy consumption $e_{\Sigma }$	0.488	0.496	0.512	0.526	**0.519**	0.540	0.551	0.568	0.525
	Total collision time $t_{\Sigma }^{colli}$	**0.000**	**0.000**	**0.000**	**0.000**	**0.000**	**0.000**	**0.000**	**0.000**	**0.000**
	Route balance index $d_{Rv}^{balan}$	**0.000**	0.670	0.550	0.489	0.452	0.467	0.433	0.422	0.435
	Solution time (s)	**409**	**402**	424	431	**428**	**433**	467	475	433.625
PSO	Makespan $t_{\Sigma }$	0.517	0.398	0.342	0.250	0.245	0.186	0.189	0.174	0.288
	Total energy consumption $e_{\Sigma }$	0.499	0.507	0.524	0.528	0.536	0.554	0.563	0.580	0.536
	Total collision time $t_{\Sigma }^{colli}$	**0.000**	**0.000**	**0.000**	**0.000**	**0.000**	**0.000**	**0.000**	**0.000**	**0.000**
	Route balance index $d_{Rv}^{balan}$	**0.000**	0.662	0.537	**0.476**	0.459	**0.430**	0.418	0.420	0.425
	Solution time (s)	410	429	436	443	447	451	**445**	472	441.625
DQN	Makespan $t_{\Sigma }$	**0.475**	0.371	0.319	**0.223**	0.227	0.183	0.182	0.160	0.268
	Total energy consumption $e_{\Sigma }$	0.483	0.519	0.517	0.521	0.525	**0.526**	0.552	0.564	0.526
	Total collision time $t_{\Sigma }^{colli}$	**0.000**	**0.000**	**0.000**	**0.000**	**0.000**	**0.000**	**0.000**	**0.000**	**0.000**
	Route balance index $d_{Rv}^{balan}$	**0.000**	0.655	**0.514**	0.483	0.460	0.451	0.428	**0.407**	0.425
	Solution time (s)	1842	1977	2085	2114	2026	2129	2288	2163	2078.000

## Data Availability

Data are contained within the article.
